# Engineered phage with antibacterial CRISPR–Cas selectively reduce *E. coli* burden in mice

**DOI:** 10.1038/s41587-023-01759-y

**Published:** 2023-05-04

**Authors:** Yilmaz Emre Gencay, Džiuginta Jasinskytė, Camille Robert, Szabolcs Semsey, Virginia Martínez, Anders Østergaard Petersen, Katja Brunner, Ana de Santiago Torio, Alex Salazar, Iszabela Cristiana Turcu, Melissa Kviesgaard Eriksen, Lev Koval, Adam Takos, Ricardo Pascal, Thea Staffeldt Schou, Lone Bayer, Tina Bryde, Katja Chandelle Johansen, Emilie Glad Bak, Frenk Smrekar, Timothy B. Doyle, Michael J. Satlin, Aurelie Gram, Joana Carvalho, Lene Jessen, Björn Hallström, Jonas Hink, Birgitte Damholt, Alice Troy, Mette Grove, Jasper Clube, Christian Grøndahl, Jakob Krause Haaber, Eric van der Helm, Milan Zdravkovic, Morten Otto Alexander Sommer

**Affiliations:** 1 SNIPR BIOME ApS, Copenhagen, Denmark; 2 JAFRAL, Ljubljana, Slovenia; 3 JMI Laboratories, North Liberty, IA USA; 4 Division of Infectious Diseases, Weill Cornell Medicine, New York City, NY USA; 5 Novo Nordisk Foundation Center for Biosustainability, DTU Biosustain, Kongens Lyngby, Denmark

**Keywords:** Drug discovery, Drug development, Preclinical research, Molecular biology

## Abstract

Antibiotic treatments have detrimental effects on the microbiome and lead to antibiotic resistance. To develop a phage therapy against a diverse range of clinically relevant *Escherichia coli*, we screened a library of 162 wild-type (WT) phages, identifying eight phages with broad coverage of *E. coli*, complementary binding to bacterial surface receptors, and the capability to stably carry inserted cargo. Selected phages were engineered with tail fibers and CRISPR–Cas machinery to specifically target *E. coli*. We show that engineered phages target bacteria in biofilms, reduce the emergence of phage-tolerant *E. coli* and out-compete their ancestral WT phages in coculture experiments. A combination of the four most complementary bacteriophages, called SNIPR001, is well tolerated in both mouse models and minipigs and reduces *E. coli* load in the mouse gut better than its constituent components separately. SNIPR001 is in clinical development to selectively kill *E. coli*, which may cause fatal infections in hematological cancer patients.

## Abstract (web summary)

Phage engineered with tail fibers and CRISPR–Cas reduce *Escherichia coli* load in animals.

## Main

Chemotherapeutic regimens used to treat hematological malignancies cause bone marrow suppression and gastrointestinal mucositis with associated increased intestinal permeability^1–4^. Translocation of gut bacteria, including *Escherichia coli*, from the gastrointestinal tract is a frequent cause of bloodstream infections^5^. The mortality related to bloodstream infections caused by enteric bacteria such as *E. coli* is 15–20%^6^; to decrease the chance of infection, antibiotics may be given before treatment in people at risk of low numbers of neutrophils in the blood^7^. Fluoroquinolones are used off-label in the United States, based on the results of two randomized trials demonstrating a decrease in bacterial infections in immunocompromised patients after use^7–9^. Fluoroquinolones have side effects, and their use in oncology patients has been accompanied by rising bacterial resistance^10^. In immunocompromised patients with hematological malignancies who develop chemotherapy-induced neutropenia, *E. coli* is responsible for 25.1–30% of all bacteremia cases^11,12^ and up to 65% of *E. coli* isolated as the causative pathogen from bloodstream infections in patients with hematological cancers undergoing hematopoietic stem cell transplantation were resistant to fluoroquinolones^13^. Other clinical options are needed that would prevent infections in these vulnerable patients, especially fluoroquinolone-resistant *E. coli*.

Bacteriophage therapy has been used before the broad availability of antibiotics^14^, but has now regained interest^15^ due to the rise in bacterial antimicrobial resistance combined with several successful individual case reports^16–18^. Still, few clinical trials with wild-type (WT) phages have been conducted^19–22^ and, although several have been directed toward *E. coli*, these have failed to produce convincing results in larger randomized controlled trials likely due to incomplete coverage of the target strains by the phage cocktail^23^. Recent efforts have used large-scale systematic screening of phages to broadly cover target strains, including characterization of phages (*n* = 41) targeting *Klebsiella pneumoniae* strains (*n* = 17)^24^ and phages (*n* = 248) targeting *Vibrio* strains (*n* = 294)^25^. T3 phage tail fibers have also been engineered to augmenting the spectrum of strains targeted by the engineered phage^26^. Finally, CRISPR–Cas systems can contribute to targeting efficacy as a complementary killing modality to the lytic activity of the phage. CRISPR–Cas complexes in some systems can bind to a homologous DNA target sequence and result in DNA degradation^27,28^. Because prokaryotes lack error-prone nonhomologous end-joining and rely on homologous recombination to repair DNA damage, they are prone to cell death following DNA degradation. This vulnerability has been exploited by using CRISPR–Cas as an antimicrobial modality for several bacteria, including *Staphylococcus aureus, E. coli* or *Clostridioides difficile*^29–35^.

**Fig. 1:**
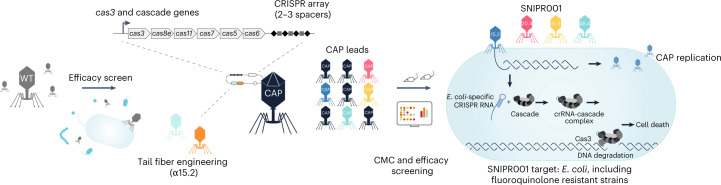
An overview of the SNIPR001 creation process. First, WT phages are screened against a panel of *E. coli* strains. Then, phages with broad activity against *E. coli* are tail fiber engineered and/or armed with CRISPR–Cas systems containing sequences specific to *E. coli*, creating CAPs. These CAPs are then tested for host range, in vivo efficacy and CMC specifications. SNIPR001 comprises four complementary CAPs and is a new precision antibiotic that selectively targets *E. coli* to prevent bacteremia in hematological cancer patients at risk of neutropenia.

To address the significant unmet medical need for new prophylactic agents for patients with hematological malignancies, we develop SNIPR001, which is a combination of four CRISPR–Cas-armed phages (CAPs) that specifically target a diverse spectrum of *E. coli* strains. Our research process for designing SNIPR001 includes several steps (Fig. 1). In short, a library (*n* = 162) of WT phages was tested in vitro on a panel of phylogenetically diverse *E. coli* strains representing the biology of the target bacterium *E. coli*. WT phages with the broadest and most complementary target strain coverage were selected for further engineering. Selected WT phages were subjected to both tail fiber engineering and CRISPR–Cas arming to create a library of CAPs. The CAP library was assessed for manufacturability, in vitro stability, spectrum of efficacy, in vivo pharmacokinetics and efficacy. A combination of four CAPs was selected to create the development candidate SNIPR001, which has now entered clinical development (ClinicalTrials.gov ID NCT05277350).

## Results

### Phage screening

**Fig. 2:**
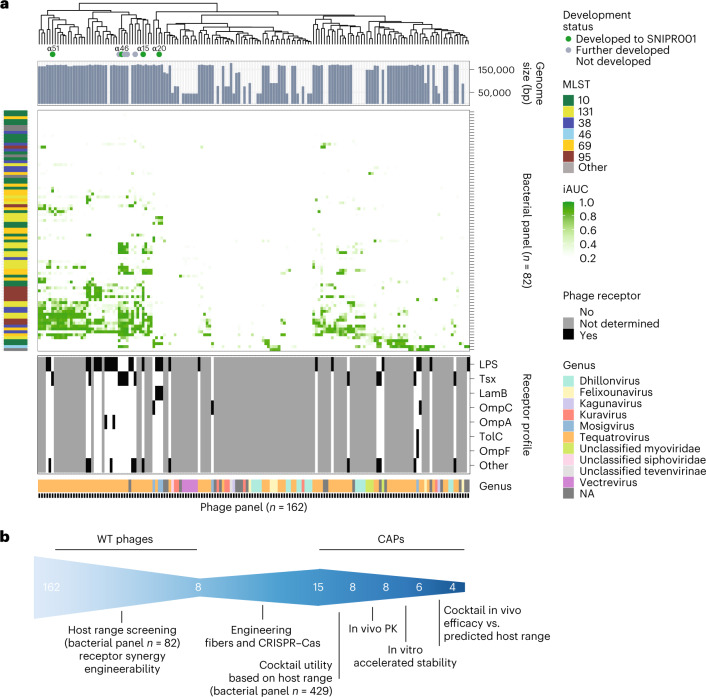
Wild type phage screening and development funnel. **a**, Heatmap showing the potency of 162 WT phages (horizontal axis) against 82 *E. coli* strains (vertical axis) based on growth kinetics. iAUC values are shown as a gradient of green. Phage genome size based on the sequencing data is shown in the top bar graph. For a selected group of phages, the cognate bacterial receptor protein was determined and shown in the bottom panel. The phage taxonomical classification based on the sequencing data is annotated in the bottom bar. The top tree shows the relationship between the phages based on their growth kinetics. The eight phages that were selected for engineering (α15, α17, α20, α31, α33, α46, α48 and α51) are highlighted by circles, four of those (α15, α20, α48 and α51) that form the basis of SNIPR001 are colored green. **b**, Overall development funnel of SNIPR001 starting with the 162 WT phages and, after engineering and selection assays, resulting in final cocktail of four CAPs in SNIPR001 with per CAP details described in Extended Data Table 1a.

For the development of SNIPR001, we initially screened a library of 162 lytic phages derived from wastewater, phage banks and commercial phage cocktails (Supplementary Table 1). The host range and potency of the phages were assessed by a stringent in vitro growth kinetics assay against either an internal panel of 429 phylogenetically diverse *E. coli* strains, or an abbreviated panel of 82 *E. coli* strains (Fig. 2a), selected to adequately represent the full 429 strain panel. The *E. coli* strains originated from patients with bloodstream infections^5^ and urinary tract infections, from feces of humans with no known disease, and from the *E. coli* reference collection^36^. For a subset, we determined their receptors using efficiency of plating (EoP) assays on two broadly sensitive strains, their deep-core (∆*rfaD*) lipopolysaccharide (LPS) mutant derivatives and their surface protein knock-out mutants (Tsx, LamB, OmpC, OmpA, TolC and OmpF) from thereof (Supplementary Fig. 2). Based on the results, the eight phages α15, α17, α20, α31, α33, α46, α48 and α51 (all members of *Tevenvirinae)* were selected on their orthogonal and broad-spectrum effect, complementary binding to bacterial surface receptors, as well as engineerability to stably carry inserted cargo (Fig. 2b).

### Tail fiber engineering

**Fig. 3:**
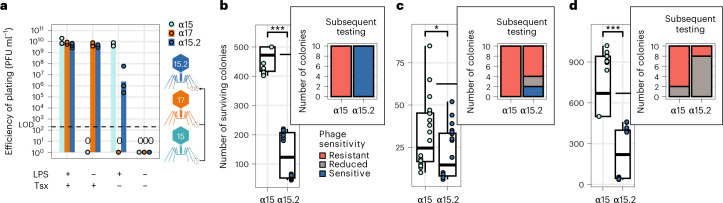
Tail fiber engineering. **a**, EoP results of LPS-dependent WT α15, Tsx-dependent WT α17 and engineered CAP α15.2 that consolidates both WT phages’ receptors. Presented titers (PFU ml^−1^) were obtained from independent biological triplicates as dots, with averages illustrated as bars. **b**–**d**, Lawn kill assay results of *E. coli* are shown as boxplots, whiskers indicate maximum and minimum values, box bounds indicate 25th and 75th percentile, with center line indicating the median; b1460 (**b**), b1475 (**c**), b1813 (**d**) with phages WT α15 and CAP α15.2. Significances *P < 0.05 and ***P < 0.001, *P* values (two-sided Mann–Whitney *U* test) were calculated from two independent biological duplicates comprised of ten replicates. Holm’s method adjusted *P* values are 1.59 × 10^−7^, 3.36 × 10^−2^ and 1.6 × 10^−7^ for b1460, b1475 and b1813, respectively. Distribution of 10 EoP profiles of survivor colonies purified from WT α15 lawn kill assay (**b**–**d** insets). Resistant, no plaque formation with tested phage; sensitive, EoP similar to that of parental strain; reduced, EoP (>1–2 log_10_) lower than that of the parental strain.

We determined that α20 requires the presence of both LPS and maltoporin LamB, while the remaining selected phages were dependent on LPS or nucleoside transporter Tsx to infect their hosts (Fig. 2a). Given the dual receptor use of α20 and the conserved nature of the Tsx protein, we deemed the initial need for tail fiber engineering small for the majority of our phages. However, one of the broadest host-range phages on our test panels, α15, was solely dependent on LPS in propagation host *E. coli* b52 (Fig. 2a and Supplementary Fig. 2); and because LPS is extremely diverse and phage-resistant clones characterized by mutations in one of the many LPS biosynthesis genes can easily evolve during therapy^37^, we wanted to expand the receptor repertoire of α15. T-even phages bind to cell receptors using their long tail fibers or a monomeric adhesin that caps the distal tip of these trimeric fibers^38^. Thus, we chose a Tsx-binding adhesin from phage α17 and engineered it into α15.2 to consolidate both affinities in one phage. Virions of this phage are carrying stochastic combinations of two-receptor affinity, enabling them to infect bacterial cells via both receptors (Fig. 3a and Supplementary Fig. 2). We then hypothesized that α15.2 should select for a reduced number of resisters in comparison to the ancestor LPS-dependent WT α15. We selected clinical *E. coli* strains b1460, b1475 and b1813 where α15.2 outperformed WT α15 in the kinetic assays and subjected them to lawn kill assays. Indeed, α15.2 substantially led to a reduced number of survivors in comparison to WT α15 albeit with different levels per tested strain (Fig. 3b–d). Ten random purified colonies from WT α15 challenged group of each tested strain were as well tested for EoP with WT α15 and CAP α15.2. In accordance, results demonstrate a clear benefit of the tail fiber engineered α15.2 over LPS-dependent WT α15, as α15 survivors mostly retained sensitivity to CAP α15.2 despite being resistant WT α15 (Fig. 3b–d, insets).

### CRISPR–Cas arming of phages to target *E. coli*

**Fig. 4:**
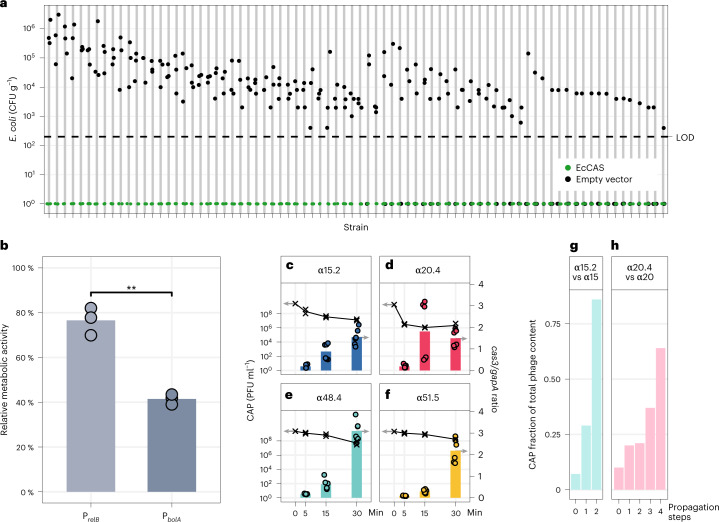
CRISPR-Cas-mediated *E. coli* elimination, activity in biofilms, and CAP competitive advantage. **a**, CRISPR–Cas-driven elimination of an abbreviated panel of 82 *E. coli* clinical isolates by conjugation of CGV-EcCAS (green) or empty vector (gray). The conjugation efficiency was determined by spotting a dilution series of the conjugation reaction on LB agar supplemented with antibiotics (*n* = 3 indicated by dots). The LOD was 200 CFU ml^−1^. **b**, Reduction of the metabolic activity of biofilms by CGVs that differed only in the promoter driving the expression of the CRISPR–Cas system, which are targeting the bacterial chromosome. Experiments were carried out in triplicate, measuring the relative metabolic activity between expression without a promoter and a given promoter. Average relative activities for P_*relB*_ and P_*bolA*_ were significantly different and illustrated as dots, with averages illustrated as bars (two-sided Student’s *t*-test, *P* = 0.0052) 83.7 ± 6.7%, and 45.3 ± 2.5%. **c**–**f**, RT-qPCR showing increasing levels of cas3 transcripts relative to housekeeping gene gapA transcripts (replicates as dots, averages as bars) in negative correlation with decreasing number of unabsorbed phages over time in a synchronized infection (replicates as crosses, averages as lines). Cas3 activity is measured as ratio of cas3 transcripts relative to gapA transcripts, and number of unabsorbed phages in PFU ml^−1^. The results shown are the average of two independent biological replicates with technical triplicates. Bars or lines, respectively, indicate average values of these replicates, with error bars indicating standard deviation. **g**, Fraction of CAP and WT phage during coculture with a host strain susceptible to both phages. CAP α15.2 increases its relative abundance compared to the WT phage from 7% to 86% over two consecutive passages. **h**, CAP α20.4 outcompeted WT α20 by increasing its relative abundance during coculture with the common target *E. coli* strain b230 from 10% to 68% over four consecutive passages. **g**,**h**, The ratio of CAP and WT phage during coculture with a host strain (*E. coli* b230) susceptible to α15.2, α15, α20.4 and α20. CAP α15.2 increase its relative abundance compared to the WT phage from 7% to 86% (**g**) while CAP α 20.4 outcompeted WT α20WT from 10% to 68% (**h**).

To CRISPR–Cas arm the selected lytic phages and generate a library of CAPs, the type I-E CRISPR–Cas system of *E. coli*^39^ was engineered (Supplementary Fig. 1) to target phylogenetically diverse *E. coli* strains. A CRISPR-guided vector (CGV-EcCas) was generated, containing the *cas3* gene (*ygcB*) and a downstream cascade gene complex encoded by *casA* (*ygcL*, *cas8e*), *casB* (*ygcK*, *cas11*), *casC* (*ygcJ*, *cas7*), *casD* (*ygcI*, *cas5*) and *casE* (*ygcH*, *cas6)*, and a CRISPR array targeting the *E. coli* genome (Fig. 1). To evaluate the killing efficiency of the CRISPR–Cas system, the CGV-EcCas was conjugated to *E. coli* strain b52, showing an average reduction of 3.5 log_10_ CFU ml^−1^, compared to the empty vector (Supplementary Fig. 3). As expected, no effect was observed after conjugating the CGV-EcCas to a nontarget *E. coli* strain (Supplementary Fig. 3). The killing efficiency of CGV-EcCas was further assessed on the abbreviated panel of 82 *E. coli* strains. Conjugative delivery of the empty vector was accomplished in 75% of the isolates (Fig. 4a). For all strains where the CGV-EcCas was delivered, bacterial counts were reduced below the limit of detection (LOD, 200 CFU ml^−1^) corresponding to a reduction of 1–6 log_10_, highlighting the potent CRISPR–Cas-mediated killing (Fig. 4a).

We aimed to engineer our CRISPR–Cas systems to be functional under restricted bacterial growth conditions, which have been observed in the gut or in biofilms^40^. We tested two relevant promoters (P_*relB*_^41^ and P_*bolA*_^42^) for their performance, both in planktonic cells grown in standard growth conditions (lysogeny broth (LB), 37 °C) and in biofilms, grown on peg lids in 96-well plates. Significant killing, measured as reduction of metabolic activity, was observed in *E. coli* biofilms when the CRISPR–Cas system was expressed from P_*bolA*_ compared to P_*relB*_ (Fig. 4b). As promoter P_*bolA*_ showed the best overall performance in the different conditions, it was chosen for transcription of the CRISPR–Cas system in the CAPs.

The eight selected WT phages were CRISPR–Cas-armed to generate 15 CAP variations (Extended Data Table 1a). In addition to promoter P_*bolA*_, the CRISPR–Cas systems were engineered to express from a synthetic constitutively expressed *E. coli* promoter (P_J23100_) to further strengthen the CRISPR–Cas expression (Supplementary Fig. 1). CRISPR arrays were designed to target multiple virulence (spacers 1, 2 and 3) or essential genes (spacers 4 and 5; Extended Data Table 1b), as targeting multiple regions has been shown to prevent resistance evolution^43^. To confirm the CRISPR–Cas activity in the CAPs, we measured the *cas3* transcripts in samples obtained at 5, 15 and 30 min following a synchronized infection with the equal multiplicity of infection (MOI) of CAP α15.2 in comparison to WT α15 using RT-qPCR and observed increasing levels of *cas3* RNA only upon CAP α15.2 infection (Supplementary Fig. 4). Next, we extended this assay to all four CAPs (α15.2, α20.4, α48.4 and α51.5) and demonstrated increasing levels of *cas3* transcripts highlighting that the CAPs expressed the CRISPR–Cas system during infection of a target strain (Fig. 4c–f).

To demonstrate the competitive superiority of the CAPs, we performed competition experiments in which CAPs (α20.4 and α15.2) and their WT ancestral phages were cocultured with *E. coli* strain b230, serving as a target for both competing phages. Approximate initial ratios of 1 CAP to 9 WT phages were cocultured and passaged four times on fresh target cells in liquid cultures. After each passage, the relative abundance of CAP and WT phage particles was evaluated. Both CAPs outcompeted their WTs within four rounds; CAP α20.4 reached 68% after four rounds and CAP α15.2 reached 86% after two rounds (Fig. 4g–h), demonstrating an improved fitness compared to the WT phages.

### Selection and characterization of the optimal CAP cocktail

The activity of the 15 CAPs was tested against the *E. coli* panel (*n* = 429) using the growth kinetics assay (Supplementary Fig. 5). The individual CAPs showed activity toward 4.1–29.4% of the strains tested. To maximize our coverage, we sought to rationally combine CAPs with a broad and complementary spectrum of activity. Thus, we made subsets of CAP cocktails based on our in silico predictions using individual performances and tested their combinatorial in vitro performance. These results showed good compliance with our predictions (Supplementary Fig. 6). The initial 15 CAPs could be classified into four clusters based on their host-range profiles (Supplementary Fig. 5). We then excluded the seven lowest-ranking CAPs based on their redundant host-range in our cocktail predictions (Supplementary Fig. 7). Thus, eight CAPs (α15.2, α15.4, α17.2, α20.4, α46.4, α48.4, α51.5 and α51.6) were chosen for further assessments. First, all eight CAPs were individually orally dosed to mice (*n* = 3) and their normalized recovery (Supplementary Fig. 8) showed that all CAPs could be retrieved from fecal matter. Next, in vitro stability was assessed at accelerated conditions (40 °C, *n* = 3). Based on these results, two CAPs (α15.4, α17.2) were deselected as their titer dropped to below 1% of the starting material (Supplementary Fig. 9). The resulting six CAPs were individually tested (*n* = 6) in a mouse efficacy model (Supplementary Fig. 10), these results were combined with the predicted host range of the simulated cocktails (Supplementary Fig. 11; *n* = 15) and verification of complementing use of surface receptors for infection, resulting in the selection of CAPs α15.2, α20.4, α48.4 and α51.5 as the optimal CAPs for SNIPR001.

The ancestors of CAPs α15.2, α20.4, α48.4 and α51.5 are classified under the *Tevenvirinae* subfamily. Specifically, α15, α48 and α51 share 96.4%, 96.6% and 96.1% sequence similarity to *E. coli* phage T2, respectively, whereas α20’s closest relative is *E. coli* phage RB69 (96.8%; Supplementary Fig. 12). In silico analyses of the genomes of SNIPR001 showed that the CAPs encode no known transposase or integrase genes, indicating that the phages are not temperate, and thus not predicted to be capable of inserting their DNA in bacterial cells. In addition, we observed no antimicrobial resistance markers or virulence genes in the phage genomes (Supplementary Table 2). We investigated whether SNIPR001 CAPs cause generalized transduction and found no evidence of transduction with the LOD of 2 × 10^−7^ for frequency of transduction (Supplementary Table 3).

### Developing a drug product from individual CAPs

Manufacturing a stable drug product comprised of four engineered phage particles requires establishing a phage and bacterial host collection, creating a Bacterial Master Cell Bank and a Master Phage Seed and turning the four resulting individual drug substances into a final SNIPR001 drug product (Supplementary Fig. 13). An important aspect of the chemistry, manufacturing and control (CMC) process is maintaining the stability of the individual components over time. We measured the titer of the individual CAPs at the stage of drug substances and found no indication of stability issues over 5 months of storage (Supplementary Fig. 14). To confirm the presence of the engineered phage parts during the CMC process, we established test criteria (Supplementary Table 2) based on whole genome sequencing of the samples. All four CAPs passed the acceptance tests, validating the presence of the CRISPR–Cas system and overall sequence identity to the CAP references (Supplementary Table 2). The final release testing criteria for the drug substances are listed in Supplementary Table 4.

### SNIPR001 does not affect other gut-associated bacteria

Ideally, a phage-based therapy should not disturb the nontargeted genera of the microbiome, thus the specificity of SNIPR001 toward *E. coli* was assessed by investigating its effects on a panel of strains, which includes non-*E. coli* species that are *E. coli* relatives, as well as a range of families associated with the commensal bacterial community in the gut bacteria (and *E. coli* as a positive control). The bacteria were cultured without CAPs, with the SNIPR001 cocktail or with individual SNIPR001 CAPs (*n* = 4). The growth in CFU ml^−1^ was evaluated over a 4-h period (ΔCFU ml^−1^_4 h–0 h_). In parallel, *E. coli* b2480 was grown under the same conditions as a positive control (Supplementary Fig. 15). We observed no significant effect (*P* > 0.05, two-sided Student’s *t*-test, FDR corrected with Holm’s method) of the SNIPR001 cocktail or any of the SNIPR001 CAPs on non-*E. coli* strains, while the growth of *E. coli* was significantly inhibited (*P* < 0.05, two-sided Student’s *t*-test, FDR corrected with Holm’s method). Thus, SNIPR001 is not expected to impact the gut microbiome beyond the target *E. coli*.

### SNIPR001 in vitro host-range in clinical target population

**Fig. 5:**
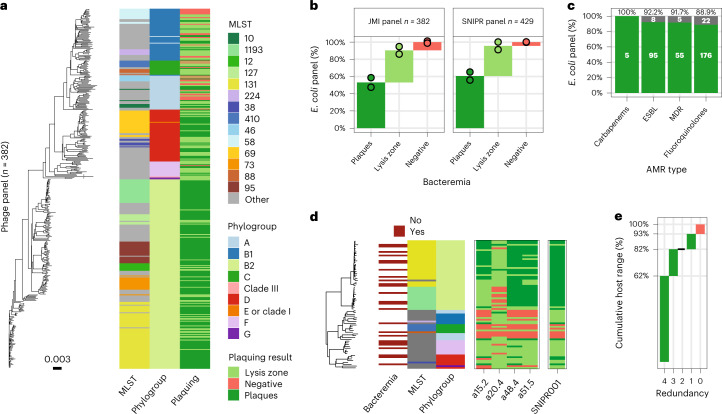
In vitro validation of SNIPR001 on clinical *E. coli* strains. **a**, An unrooted phylogenetic tree of the JMI strains displaying a clinical panel of 382 *E. coli* strains encompassing nine phylogroups and 118 MLSTs. Plaquing data reflects a single plaquing replicate. One strain, *E. coli* b4038, with a long branch (indicated by a break) has been truncated to 37% of the original length. Phylogenetic distance scale indicated below the phylogenetic tree, as computed by MASH. **b**, A spot assay was used to analyze the efficacy of SNIPR001 against the clinical panel of 382 *E. coli* strains (from JMI Laboratories) isolated from bloodstream infections and the internal 429 *E. coli* strain panel. The spot assay was conducted as two independent experiments, with bars indicating average cumulative panel fraction and dots indicating the results of each duplicate relative to prior means. **c**, Coverage of SNIPR001 does not depend on antibiotic-resistant phenotypes; consequently, SNIPR001 targets > 90% of *E. coli* strains that are carbapenem resistant, ESBL-producing or MDR, and 89% of fluoroquinolone-resistant *E. coli* strains. Numbers indicated in each green or gray bar indicate the number of bacteria susceptible or resistant to SNIPR001, respectively, for each resistance category generated from a screening of 382 strains and subset to the number of strains with a given resistance. **d**, A midpoint-rooted phylogenetic tree of the 72 fluoroquinolone-resistant *E. coli* strains isolated from fecal samples of hematological cancer patients. A total of 67 of the 72 strains are susceptible to at least one of the four CAPS in SNIPR001. Plaquing results are generated by the conservative consensus between two runs of plaquing, that is displaying the outcome with lower plaquing efficiency. **e**, Redundancy distribution showing 82% of the fluoroquinolone-resistant *E. coli* strains (*n* = 72) from **d** are targeted by at least two different CAPs.

To understand the potential effect in strains relevant to hematological cancer patients, the coverage of SNIPR001 was tested against our internal *E. coli* panel (429 strains) and a set of 382 clinical *E. coli* strains (JMI Laboratories). These JMI strains originated from patients with bloodstream infections hospitalized in hemato-oncology units across four different regions from 2018–2020 (Asia-Pacific 54 isolates, Europe 161 isolates, Latin America 26 isolates and North America 141 isolates; Supplementary Fig. 16). The genotypic distribution of *E. coli* strains in the patient population was determined using whole genome sequencing and was found to be diverse, representing nine phylogroups and 118 multilocus sequence types (MLSTs; Fig. 5a and Supplementary Fig. 17). We recorded phage infectivity against the *E. coli* panel using a spotting assays. Visible single plaques were differentiated from lysis zones in cases where single plaques could not be verified. All spotting assays were run in duplicates. We observed overall coverages of 90.4 ± 1.6% of SNIPR001 in the 382 JMI *E. coli* panel, and of 95.6 ± 0.3% of SNIPR001 on the internal *E. coli* panel (429 strains). Furthermore, we observed plaques in 53.1 ± 7.7% and lysis zones in 37.3 ± 6.1% of the JMI panel strains, and similarly, plaques in 60.1 ± 6.6% and lysis zones in 35.4 ± 6.3% of the internal panel strains (Fig. 5b). SNIPR001 showed 100% coverage in the B2 phylogroup, representing 53% of the JMI panel. This phylogroup is correlated with multidrug resistance and virulence. Additionally, we observed that SNIPR001 covered 91.7% (*n* = 55) of strains classified as multidrug resistant (MDR), 100% (*n* = 5) of carbapenem-resistant strains, 92.2% (*n* = 95) of extended-spectrum β-lactamases producing strains and 88.9% (*n* = 176) of strains that are resistant to fluoroquinolones, such as ciprofloxacin and levofloxacin (Fig. 5c).

Finally, we validated SNIPR001 on a clinical panel (*n* = 72) of fluoroquinolone-resistant *E. coli* strains that were isolated from either a fecal sample or a perianal swab from hematological cancer patients. This population represents the expected clinical target patient population being pursued (SNIPR001 has been designated fast-track status by the FDA). A subset of these strains gave rise to bloodstream infection (Fig. 5d). 82% of the *E. coli* strains (*n* = 72) were susceptible to at least two or more of the CAPs in SNIPR001, and 93% of the strains were susceptible to the whole SNIPR001 cocktail (Fig. 5e). These data demonstrate the benefit of SNIPR001 compared to the individual CAPs with regards to improving the spectrum of efficacy.

### Tolerability and recovery of SNIPR001 in minipigs

**Fig. 6:**
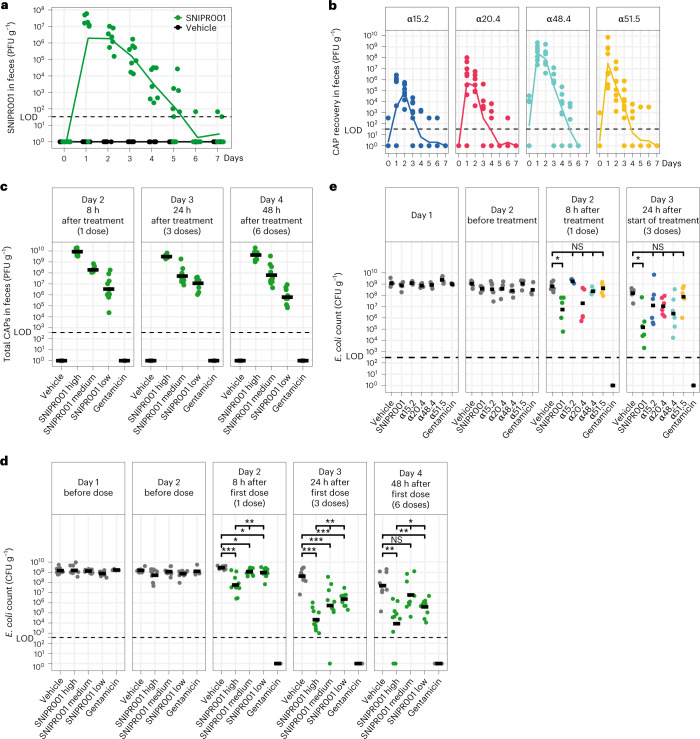
SNIPR001 in vivo evaluation in mice and minipigs. **a**, CAP recovery in minipigs feces after a single p.o. dose of 2 × 10^12^ PFU of SNIPR001 (*n* = 8, green) or vehicle (*n* = 6, gray) over 1 week with daily sampling. Trend lines indicate average recovered phage in PFU per gram feces, dots indicate individual measurement points. LOD of 33 PFU g^−1^ feces indicated by the dotted line. **b**, CAP recovery in minipig feces after a single p.o. dose of 2 × 10^12^ PFU of a single CAP (*n* = 8 minipigs received either α15.2, α20.4 or α51.5; *n* = 7 minipigs received α48.4) over 1 week with daily sampling. Trend lines indicate average recovery, while points indicate individual measurements. Recovery was measured in PFU per gram feces. LOD of 33 PFU g^−1^ feces (dotted line). **c**, CAP recovery in mouse feces 8 h, 24 h and 48 h after the start of treatment with three times daily administration of varying doses of SNIPR001 (*n* = 10 for low, medium and high, green), vehicle (*n* = 10, gray), or gentamicin (*n* = 4, gray). Recovery is measured in PFU g^−1^ feces, LOD of 371 PFU g^−1^ feces (dotted line). **d**, *E. coli* b17 recovery in mouse feces indicates increased SNIPR001 effect with increased dose; color legend and group sizes are the same as in **c**. *P < 0.05, **P < 0.01, ***P < 0.001; statistical analyses were performed using two-sided Kruskal–Wallis tests for comparison of all SNIPR001-treated groups, two-sided Mann–Whitney *U* test was used for comparison of treated groups with vehicle corrected using Holm’s method separately for each day. The exact *P* values are shown in Extended Data Table 5. Recovery is measured in CFU per gram feces, with a LOD of 371 CFU g^−1^ feces. Animals that have begun SNIPR001 treatment are indicated in green, others in gray. **e**, *E. coli* b17 recovery in mouse feces 8 h and 24 h after the start of treatment with three times daily administration of CAPs α15.2, α20.4, α48.4 or α51.5 (*n* = 6 for each CAP) and in combination as SNIPR001 (*n* = 6) confirming synergy of the CAPs, as well as vehicle (*n* = 6), and gentamicin (*n* = 3). Differences in CFU per gram tested by a two-sided Mann–Whitney *U* test, *P* values corrected with Holm’s method. Adjusted *P* values for comparisons of vehicle and SNIPR001 are both 0.022 for days 2 and 3.

The tolerability and gastrointestinal recovery of SNIPR001 were evaluated in female Göttingen minipigs. Blood and feces were sampled over 7 d following oral administration of 2 × 10^12^ PFU of SNIPR001 or vehicle. No CAPs were recovered from plasma, indicating no systemic exposure, while CAPs were recovered in the feces up to 7 d after SNIPR001 administration with a peak of 2 × 10^7^ PFU 24 h postdosing (Fig. 6a). The minipigs exhibited no clinical signs and no significant changes were observed in hematology or biochemistry parameters, in particular, no changes were seen in any immune cells (Supplementary Fig. 18), compared to vehicle treatment, supporting that SNIPR001 was well tolerated (Supplementary Figs. 19, 22–25, and Supplementary Table 5). Similar recoveries were obtained with the individual CAPs (Fig. 6b). In conclusion, SNIPR001 appears to be well tolerated in Göttingen minipigs with gastrointestinal recovery.

### Efficacy in a mouse colonization model

To assess the in vivo efficacy of the four selected CAPs in reducing *E. coli*, we adapted a mouse gut colonization model from ref. ^44^ for *E. coli* strain b17 (Supplementary Fig. 20). Streptomycin was administered for 3 d to reduce Gram-negative bacteria from the mouse gastrointestinal tract, after which streptomycin administration was stopped and animals were inoculated once perorally with *E. coli* b17 (1 × 10^7^ CFU). This allowed stable colonization for 3–4 d. Aiming at assessing the efficacy of CAPs on established colonization, treatment was started 2 d after inoculation and the study was terminated on day 4 after inoculation, as the colonization starts to drop. To ensure maximum exposure to CAPs, mice were treated with three daily doses, administered 8 h apart, for a total of six doses over 2 d.

Mice were treated by oral gavage with a high, medium or low dose (2 × 10^11^ PFU, 2 × 10^9^ PFU and 1 × 10^7^ PFU, respectively) of SNIPR001, vehicle (negative control) or gentamicin (positive control). CAP recovery in the feces ranged from 3 × 10^7^ PFU g^−1^ in the low dose to 1 × 10^10^ PFU g^−1^ in the high dose, confirming successful GI passage (Fig. 6c). These levels of CAPs were associated with a significant (*P* < 0.05, two-sided Mann–Whitney *U* test, FDR corrected) dose-dependent reduction in the target *E. coli* population compared to vehicle treated mice, after 24 h of treatment (day 3). At the high dose, SNIPR001 led to a 4 log_10_ CFU g^−1^ reduction (Fig. 6d). Despite an increased variability in bacterial recovery on day 4, possibly due to clearance of the colonizing strain as illustrated in the vehicle group, similar reductions were observed after 2 d of treatment (day 4). While the medium dose did not reach statistical significance (*P* < 0.05, two-sided Mann–Whitney *U* test), there was nevertheless a numerical reduction in comparison to the vehicle group. Subsequently, the efficacies of the individual CAPs were compared to the SNIPR001 cocktail in this model. In this experiment, a greater reduction in the colonization of the target strain was observed with SNIPR001 compared to any single CAP (which showed a numerical, but not statistically significant reduction) highlighting a benefit in efficacy from the combination (Fig. 6e). We also assayed the resistance profile of randomly sampled surviving bacteria and found no isolates that were resistant to the SNIPR001 cocktail. We did identify one isolate from one animal which was resistant to three of the four phages of the cocktail (Supplementary Fig. 21). Overall, these data demonstrate the ability of SNIPR001 to decrease the target *E. coli* in the GI tract of colonized mice.

## Discussion

Here we describe the development of SNIPR001 designed to target gut *E. coli* that frequently translocate in the bloodstream to cause bloodstream infections in patients with hematological cancers who are neutropenic. While fluoroquinolones are being used off-label, these patients continue to have high morbidity and mortality. The use of traditional antibiotics has led to significant bacterial resistance development, and the number of deaths attributable to bacterial antimicrobial resistance in 2019 has been estimated to be 1.27 million, with *E. coli* being the leading pathogen^45^. In this study, we describe the development of SNIPR001, a combination of engineered phages with the potential to address challenges related to antibiotic resistance in immunocompromised patients.

SNIPR001 combines state-of-the-art phage screening, with phage tail fiber engineering and CRISPR–Cas arming. Traditionally, phage therapy has been used experimentally with limited characterization and often applied in a highly individualized way because of the often narrow host range of individual phages^46^. Building on recent advances in phage engineering that have enabled the manipulation of virulent phages^47^ and the ability to engineer tail fibers^26^ and CRISPR–Cas arm the phages, we enhanced the potency of the phages comprising SNIPR001 to enable it to target a broader range of clinically relevant *E. coli*, including strains that are resistant to current therapies.

To deliver a development candidate ready for clinical testing, we established a traceable manufacturing process resulting in stable CAP substances, and final confirmation of the efficacy of SNIPR001 on large and clinically relevant strain panels supports the clinical potential of the SNIPR001 cocktail. The observed 4 log_10_ reduction of *E. coli* in our in vivo model is a clear improvement over the previous studies^35,48^.

SNIPR001 is an orthogonal antimicrobial approach as it has shown activity in MDR strains. In addition, there is emerging evidence that maintaining a normal microbiome is important for upholding immunological tonus and potentially benefiting the outcome of oncology treatments^49^, and this has also been recognized in the most recent guidance on prophylactic management of patients at risk of febrile neutropenia^7^. In this context, in vitro studies with SNIPR001 have shown specificity toward *E. coli* with no off-target effects toward any of the tested non-*E. coli* strains, thereby having a less detrimental effect on the microbiome. In the future, individualized combinations of narrow-spectrum antibiotics such as SNIPR001 may be used first-line rather than use in addition to broad-spectrum antibiotics such as fluoroquinolones.

As with any nonclinical study, the translatability of the in vitro and preclinical findings into humans requires investigation, in particular for MDR strains. Although we did not observe structural resistance toward SNIPR001 in mice, resistance development, and the synergy that a combination of CAPs provides, are challenging to study in vivo with a complex drug product like SNIPR001. Furthermore, part of the activity spectrum of SNIPR001 is driven by lysis zone formation and not plaquing, and it is to be investigated how this phenotype translates into clinical efficacy. Therefore, a clinical study to evaluate the ability of SNIPR001 to ascertain safety and its ability to reduce *E. coli* in the gut without perturbing the overall gut microbiome is currently ongoing in the United States (NCT05277350). SNIPR001 exemplifies a potentially significant therapeutic advance in the field of antimicrobials for high-risk patient populations and can serve as a blueprint for narrow-spectrum therapies for other life-threatening antimicrobial-resistant pathogens in high-risk patient populations.

## Methods

### Phage collection and isolation procedures

The starting point for the phage screening was a collection of 162 lytic WT phages, 82 were isolated in-house from commercial cocktails and environmental sources, 71 phages were obtained from a phage bank (LyseNTech, Korea) and two phages from ATCC, one phage was donated by the University of Copenhagen and six were obtained from Kirikkale University, Turkey^50^ (Supplementary Table 1). Phage isolation was carried out by using *E. coli* strain panels (see *E. coli* panels and isolation procedures). In brief, 100 μl of overnight cultures of each *E. coli* strain were mixed with 100 μl of each phage cocktail or wastewater sample. Following 6 min incubation at room temperature (in this period infection should occur), 3 ml of prewarmed top agar containing Ca^2+^ were added to the *E. coli*/phage or wastewater mixtures and poured immediately on an LB plate. Alternatively, tenfold dilutions of each cocktail were spotted on lawns prepared with isolation strains. After drying, plates were incubated at 37 °C overnight. Plaques were picked from each plate and resuspended in 500 μl of SM buffer, vortexed and stored at 4 °C. Tenfold dilutions were spotted on the isolation strain which the plaque was originally picked from. To increase the likelihood of obtaining plaques corresponding to single phages, the procedure was repeated at least three times. Lysates were prepared from single plaques picked at the previous round of propagation, DNA was extracted and their genomes were sequenced.

### *E. coli* panels and isolation procedures

Three *E. coli* panels, one internal SNIPR Biome panel and two clinically relevant panels were included in this study. The internal panel consists of 429 phylogenetically diverse *E. coli* strains, isolated from the blood of patients with bloodstream infections and urinary tract infections, from feces of humans with no known disease, animals and the environment. The strains cover seven different phylogroups (A, B1, B2, C, D, E and F), 114 MLST groups, serotypes (K- and O-type), antibiotic resistance profiles and different geographical locations of isolation.

The JMI panel comprises 382 strains *E. coli* clinical collection obtained from JMI Laboratories. These strains were isolated from patients with bloodstream infections hospitalized in hematology and oncology units across four different regions (Asia-Pacific 54 isolates, Europe 161 isolates, Latin America 26 isolates and North America 141 isolates), sourced through the SENTRY Antimicrobial Surveillance Program (2018–2020), which is composed of a network of more than 150 medical centers in more than 28 countries worldwide (https://www.jmilabs.com/sentry-surveillance-program).

Finally, the panel comprising 72 fluoroquinolone-resistant *E. coli* strains is isolated from either fecal samples or perianal swabs of hematological cancer patients hospitalized for hematopoietic cell transplantation^51,52^.

*E. coli* strains were cultivated at 37 °C in LB at 250 rpm in liquid media or on agar plates containing 1.5% (wt/vol) agar. When necessary, cultures were supplemented with ampicillin (100 μg ml^−1^), kanamycin (50 μg ml^−1^), gentamicin (15 μg ml^−1^) or amikacin (50 μg ml^−1^). All media for the growth of conjugation donor *E. coli* JKE201 (ref. ^53^) and its derivatives were supplemented with 1,6-diaminopimelic acid (80 μg ml^−1^) to complement their auxotrophy.

Both *E*. *coli* strain b52, which was used to produce α15.2, α48.4 and α51.5, and *E. coli* strain b2479, which was selected to produce α20.4, belong to phylogroup A. Strain *E. coli* b17 was used as colonizing strain in the in vivo efficacy models as the strain is susceptible to all SNIPR001 CAPs and is part of the SNIPR Biome strain bank.

### Phage screening by growth kinetics

In vitro susceptibility of the internal *E. coli* panel (*n* = 429) to the 162 WT phages was evaluated using a growth kinetics assay. The assay measures the metabolic activity of a bacteria by tracking the reduction of a tetrazolium dye to a purple compound that aggregates during bacterial growth. The colorimetric reading was recorded every 15 min over a 24-h period by using the OmniLog (Biolog)—adapted from ref. ^54^. The inhibitory area under the curve (iAUC) was calculated from the kinetic curves over the course of the experiment and was defined as the ratio between the normalized AUC of the phage-treated bacterial growth curve and the bacteria-only control. Susceptibility was defined at iAUC values ≥0.2. Prescreening, including 48 phages, was carried out at MOI 10, after which 114 phages were screened at MOI 1.

### Calculation of bacterial growth inhibition using iAUC

The growth inhibitory effect of SNIPR001 was determined using growth kinetic curves constructed using the OmniLog apparatus. To limit technical variability in measurement between timepoints, a cubic smoothing spline function was applied to the data in Scala using the ‘umontreal.ssj.functionfit’ package. To identify appropriate *ρ* and weight variables, every combination of *ρ* and weight 0.1 and 0.5 was applied in 0.1 increments (that is, 0.1, 0.2, … 0.5). The spline with the lowest mean absolute error was chosen for area under the curve (AUC) calculation. The initial cumulative amount of fluorescent dye at the initial timepoint varies slightly from well to well, leading to artificial inflation of the AUC of certain wells. Using the best smoothed square spline, the mean signal for the first 1.5 h, before any measurable growth, was removed from all growth curves to approximate a zero-growth signal intercept. The total iAUC was calculated as the sum of the Riemann midpoint sums for each timepoint along the smoothed square spline. Lastly, we calculated the iAUC as iAUC = 1 − AUC_Sample_/AUC_Control_, where AUC_Sample_ is the AUC of the spline created by a given bacteria and SNIPR001, while AUC_Control_ refers to the AUC of the spline created with a given bacteria without a given phage or CAP, or a combination of those. Thus, iAUC values usually lie between 0 and 1, where 0 indicates no growth inhibition and 1 indicates complete growth inhibition. Some biological and technical noise does result in iAUC values outside these bounds on occasion but is considered negligible.

Host range was calculated as the fraction of a panel that had an iAUC < 0.2 for each repeat. Reported standard deviations were calculated as the deviance in the number of strains with an iAUC < 0.2, and then normalized to the size of the panel, by dividing the s.d. with the size of the panel.

### Combination complementarity prediction

Phage and CAP complementarity were evaluated in silico under the assumption of complementarity—if at least one CAP in a combination of phages can strongly inhibit a given bacterial strain, the combination of CAPs is assumed to strongly inhibit that bacterial strain. In in vitro studies, the total host range was estimated by calculating the fraction of a panel that was inhibited by one or more of the members of a given CAP or phage combination. In OmniLog screenings, a strain was considered inhibited if the iAUC of phage was above 0.2 compared to control. When using plaquing results, a strain was considered inhibited if a plaque or lysis zone was observed.

In in vivo studies, the effect of CAP combinations was considered complementary, and the efficacy of individual CAPs was assessed as the log_10_-transformed difference in CFU per gram between a vehicle and a given CAP. The predicted effect of a combination was thus evaluated as the sum of these log_10_ reductions for each member of a combination.

### In silico marginal host-range calculation

To get an overview of the ability of a CAP to participate in an efficacious CAP combination, we evaluate the marginal host ranges for each CAP. The marginal host range is a measure of the gained host range when a given CAP is incorporated in a combination. This is calculated as the difference in host range between a combination with and without a given CAP of interest. By calculating the marginal host ranges of each combination for each CAP, we can compare the different CAPs with regard to their utility in adding host ranges. However, the composition of the CAP panel can lead to unfair scoring—the addition of a CAP to a combination, where one of the composing CAPs has a very similar inhibitory profile, would have an unfairly low marginal host range. Similarly, if a CAP is added to a combination of CAPs that all have very similar inhibitory profiles, the marginal utility gain would be unfairly high. If the set of CAPs being screened does not equally represent different types of inhibitory profiles, some CAPs will have misleading marginal host-range distributions. To avoid this issue, we do not generate combinations of CAPs that contain multiple CAPs that originate from the same WT phage.

To identify CAPs whose marginal host range tended to be good, we used the mode to differentiate the CAPs. The mode of the distribution for each phage was used to calculate the overall utility of phage using the density() function in R v. 4.1.0.

### Engineering phages with a CRISPR-Cas system

Phages were CRISPR–Cas armed by using homologous recombination. We inserted the payload in the region between the *pin* (encoding the inhibitor of host Lon protease) and *vs.7* (encoding a conserved hypothetical protein) gene. Recombination was carried out in bacterial cells during phage propagation. Cells carried a plasmid that served as a recombination template. Recombination template plasmids carried the sequences that were aimed to be inserted into the phage genome between ~200 bp and 700 bp flanking sequences that were homologous to the phage sequences at the insertion site. For each phage, we inserted the type I-E CRISPR–Cas system endogenous to *E. coli* (Genbank CP032679.1), that is, the *cas3* gene (*ygcB*) and the downstream genes encoding the cascade complex, *casA* (*ygcL*), *casB* (*ygcK*), *casC* (*ygcJ*), *casD* (*ygcI*) and *casE* (*ygcH)*, as well as a CRISPR array targeting selected *E. coli* sequences. For all CAPs selected, the *cas* genes originating from *E. coli* are identical. Insertion of the CRISPR–Cas system resulted in the deletion of ~7 kbp deletion of phage DNA in the *pin* - *vs.7*. The sequences of the resulting CAPs were verified by NGS (BaseClear).

### Transduction of CGVs in biofilms

*E. coli* b52 cells were grown in 96-well plates, and biofilms were allowed to develop on peg lids. Each well contained 180 µl M9 medium (Sigma-Aldrich, M6030) supplemented with 20 mM glucose, 2 mM MgSO_4_, 0.1 mM CaCl_2_, 0.1% Amicase (Sigma-Aldrich) and 0.1% mannitol. Wells were inoculated with 1 µl of overnight b52 culture. The peg lid was inserted, and the microtiter plate was incubated statically for 24 h at 37 °C. Next, the peg lid was transferred to a new plate with fresh media without washing, and the plate was incubated for an additional 24 h. After incubation, a new plate was prepared with 100 µl media and 100 µl of CGV transducing particles (~10^8^ particles) in each well (three replicates). Biofilms grown on the pegs were rinsed three times in sterile H_2_O (200 µl) before transferring them on the new plate. The plate was incubated statically for 5 h at 37 °C.

To assay the metabolic activity of cells in the biofilms, lids were rinsed three times in sterile H_2_O (200 µl) before placing them in a plate with 20 µl Alamarblue stain (Thermo Fisher Scientific) and 180 µl media in each well. Plates were incubated for 1.5 h at 37 °C and moved to a microplate reader (Synergy H1, Biotek). Fluorescence (excitation, 560 nm; emission, 590 nm) and absorbance (600 nm) were recorded for each well.

The metabolic activities of the biofilms treated with CGVs carrying one of the promoters (P_*relB*_ or P_*bolA*_) were reported relative to the metabolic activities of biofilms treated with a CGV not carrying a promoter transcribing the *cas* genes.

### Plasmid and strain construction

To construct CGV-EcCas, *cas3* and cascade genes from *E. coli* were amplified and cloned into a ColE1-type plasmid, pZE21 (ref. ^55^), containing kanamycin, gentamycin and amikacin resistance markers, and oriT RP4.

DNA fragments encoding a 3-spacer array targeting genes in *E. coli* were synthesized as gBlock fragments (IDT) flanked by AarI restriction enzymes (gB149, gB150, gB152 and gB153; Supplementary Table 6). Similarly, constitutive promoter J23100 (ttgacggctagctcagtcctaggtacagtgctagc) was synthesized as a gBlock fragment (IDT) (gB-d2; Supplementary Table 6) to drive the expression of the CRISPR array. The array contains nucleotides from the genome of *E. coli* per target locus separated by direct repeats. The protospacer adjacent motif is located adjacent to the selected target sequences in the genome of *E. coli*.

*cas3* and cascade genes from *E. coli* were amplified with primers containing *Aar*I restriction sites (TH556 and TH558; Supplementary Table 6). Similarly, pM0 constitutive promoter to drive the expression of the *cas* genes (ggattaacaatataagctgaccttcaagtattgaat) was amplified with primers TH402 and TH403 (Supplementary Table 6). To combine *cas3* and cascade genes with the CRISPR array, all plasmids were digested with BsaI and ligated with T4 DNA ligase. Finally, to generate CGV-EcCas, the CRISPR–Cas system was moved into conjugative plasmid pZE21 by InFusion HD cloning using primers TH712 to TH715 (Supplementary Table 6).

### Transformation assays

Overnight cultures were diluted (1:100) in fresh LB medium and grown to mid-exponential phase (OD_600_ ≈ 0.6). Subsequently, cells were prepared for electroporation and concentrated 50-fold in ice-cold MilliQ water. Cells were then electroporated with appropriate plasmids, allowed to recover for 1 h at 37 °C in super optimal broth, and plated on LB plates supplemented with antibiotics.

### Conjugation assays

Conjugation experiments assessing the transfer and killing efficiency of CGV-EcCas were established using *E. coli* JKE201 as the donor and *E. coli* clinical isolates as recipients (including target and nontarget and *E. coli* strains as controls). Plasmids were conjugated into *E. coli* recipients by liquid mating. Briefly, overnight cultures were diluted (1:100) in fresh LB medium, grown to OD_600_ ≈ 0.4, washed, and suspended in fresh LB to OD_600_ ≈ 0.25. 125 μl of donor and 25 μl of recipient cell suspensions were mixed for 5:1 mating in a 96-well microplate and incubated for 16 h at 37 °C. The conjugation efficiency was determined by plating a dilution series of conjugation reactions onto LB agar supplemented with antibiotics (to select for the transconjugants). The specific killing efficiency was quantified by plating 90 μl of the conjugation reactions on selective plates. The CGV-EcCas plasmid encodes kanamycin, gentamycin and amikacin resistance to enable selection for transconjugants. Viability was calculated by counting CFUs on the plates, and data were recorded as viable cell concentration (CFU ml^−1^).

### Synchronized CAP infection and *cas3* expression assay

An overnight culture of the test strain in LB was 100-fold diluted and incubated to stationary phase in LB at 37 °C with shaking, and 10-ml aliquots were subsequently separated into 50-ml falcon tubes. Each aliquot was then seeded with 50 µl of high-titer lysate of the individual CAPs, and incubation was continued under the same conditions. Additionally, a mock 10 ml LB volume for each CAPs was also seeded with 50 µl of CAP lysates and used for 0 min phage enumeration. At 5 min, 15 min and 30 min postseeding, aliquots were collected for total RNA extraction and phage enumeration. Phage enumeration aliquots were syringe filtered (0.2 µm, Sartorius AG) and subjected to an EoP assay. For total RNA extraction, 1 ml aliquots of individual cultures were centrifuged at 13.3k*g* using a table-top centrifuge for 15 s, and supernatants were discarded. Then, pellets were immediately resuspended in cold RNA Later (Thermo Fisher Scientific, AM7020) and stored at −20 °C until extraction. Total RNA was extracted using a GeneElute Total RNA kit (Sigma-Aldrich) following the manufacturer’s protocol for extraction of RNA from bacteria. After the first elution, 1 µl of Dnase I (1 U µl^−1^) was added and incubated overnight at 37 °C. The reaction was terminated by incubation at 70 °C for 15 min. The RNA was re-purified on a GeneElute column and eluted in 35 µl of kit elution buffer. Total RNA concentration was estimated on a NanoDrop instrument (Thermo Fisher Scientific, One/OneC), and 0.5–2 µg of RNA was added to a cDNA synthesis reaction containing SuperScriptIII RT enzyme (Thermo Fisher Scientific) and random decamers to prime synthesis in a 20-µl reaction volume. The cDNA reaction was diluted to 100 µl in water. RT-PCR was conducted in triplicate using 5 µl of cDNA as template, 10 µl of Power SYBR Green PCR Master mix (Thermo Fisher Scientific) and 0.2 µM of each PCR primer. PCRs were performed on an AB QuantStudio5 system (Applied Biosystems) using the standard two-step thermocycling protocol for Power SYBR Green PCR Master Mix with 60 °C annealing/extension. The forward and reverse primers for *gapA* (reference gene) were 5′-cgctaacttcgacaaatatgctggc-3′, and 5′-aggacgggatgatgttctgggaa-3′, and for *cas3* were 5′-caagtatgctaccaacggctaaag-3′ and 5′- ccaatcaaaatcaacgtcgagtga-3′. Single PCR products were confirmed for these primer pairs by melting curve analysis. Relative levels of transcripts were estimated using tenfold dilutions of purified PCR products as standards, and values were expressed as the ratio of *cas3* to *gapA* transcripts.

### Phage competition assay

Lysates of the two phages were mixed at 9:1 (WT:CAP) ratio and the phage mixtures were added to 10 ml 2xYT medium containing 10 mM CaCl_2_ and 20 mM MgCl_2_, and 100 µl overnight of *E. coli* strain b230, serving as a target for both competing phages. After 2 h incubation in a 37 °C shaking incubator, the cultures were centrifuged and 1 µl of the supernatant was added to a new b230 culture. The same steps were repeated twice.

The ratio of phages was assessed by PCR with three primers, resulting in two specific products, one for the WT phage and one for the CAP (α15/15.2—5′-ttcattgcgtatttgtagatgaagctc-3′, 5′-cttttcagacttatcttgcgtttcttaagaagttctacaagttct-3′, 5′-gtacgactgattgatcccaccagc-3′; α20/20.4—5′-atggcttttattgctaccgggt-3′, 5′-aaatctagagcggttcagtactcaaggaaatcatcccagaaactc-3′, 5′-tgctatctttggctccactgtgat-3′). PCR products were separated on a 1% agarose gel and DNA bands were stained by SYBRsafe and visualized and quantified by the ChemiDoc XRS + System (model 1708265, Bio-Rad). The background-corrected intensity of the band corresponding to the WT phage was divided by the intensity of the band corresponding to the CAP in the same lane, to obtain the ratio of the two band intensities (WT/CAP). The fraction of CAP compared to the total phage content (WT + CAP) was determined based on the calibration curve, which was made by using a set of different mixtures of the two phages and fitting a curve to the measured band intensity ratios (WT/CAP). The estimated error of the reported values is less than 20%.

### Lawn killing assay

An overnight culture of the test strain in LB was adjusted to 10^9^ CFU ml^−1^. Hundred µl aliquots of CFU ml^−1^ adjusted strain was mixed with 100 µl of 10^9^ PFU ml^−1^ to achieve a multiplicity of infection of 1 of either CAP α15.2 or WT α15 in 15 ml falcon tubes, mixed with 3 ml of molten and pretempered top agar and spread on LB plates. After lawns were solidified, plates were incubated at 37 °C overnight, and the total number of surviving colonies was counted for CAP α15.2 or WT α15 groups the next day. Assays were performed as independent biological duplicates where each experiment comprised ten technical replicates. Statistical significance was established using both replicates using a two-sided Mann–Whitney *U* test.

### Generalized transduction assay

The transduction ability of each CAP was evaluated via the generalized transduction assay. Briefly, transducing lysates were prepared by propagating each CAP on *E. coli* MG1655 *lamB*::*Cm*. This strain was modified from the WT MG1655 (700926, American Type Culture Collection) to carry a chloramphenicol selection marker. Experiments were conducted in parallel with the well-characterized lytic T4 phage (negative control), and its transducing mutant T4GT7 (ref. ^56^; positive control). Following this step, the WT *E. coli* MG1655 strain was infected at an OD_600_ of 0.3 with each transducing lysate at MOI of 0.5, 0.1 and 0.01, and spread on LB plates containing chloramphenicol. Next day, the number of transductant colonies was recorded for each CAP and control and different MOIs. The frequency of transduction was calculated as the number of transductants divided by the titer of the transducing lysate.

### Sequence analysis of CAPs

Sequences of the individual SNIPR001 CAPs were analyzed for the presence of antibiotic resistance, virulence genes and lysogeny associates genes (transposases and integrases) using databases (Extended Data Table 2). Furthermore, for release criteria during the CMC process (Supplementary Table 2), phage samples were analyzed using whole genome sequencing. This typically results in >1000× coverage of the whole phage genome. Assemblies are constructed by down-sampling the data to 1000× average coverage for the phage and assembling using SKESA. To detect differences between samples and to detect nonmajority mutations the raw reads were mapped back to the assembly using BWA (version 0.7.17).

### Phage specificity assay using liquid killing assay

SNIPR001 CAPs (α15.2, α20.4, α48.4 and α51.5) and SNIPR001 killing specificity were evaluated via a biopotency assay against a panel of human-relevant, aerobic (*n* = 6) and anaerobic (*n* = 3) bacterial strains. An *E. coli* strain b2480 was included as a positive control for phage-mediated killing (Extended Data Table 3).

In brief, overnight cultures were adjusted to 10^6^ CFU ml^−1^ in LB broth. SNIPR001 CAPs or SNIPR001 (in which each CAP was combined in equal ratio) were added at an MOI of 1 before incubation for 4 h. Untreated bacteria were cultured in parallel as controls for bacterial growth. CFU counts were recorded at 0 h and 4 h post phage treatment, and data are represented as Δlog_10_ CFU ml^−1^ by subtracting the initial inoculum (0 h) from the assay endpoint CFU per milliliter (4 h).

### CMC

The in vitro stability of phages was assessed by following the potency of CAPs in the formulation buffer overtime at 2–8 °C and at accelerated temperature (40 °C). Polypropylene cryovials were filled with one milliliter of each phage for storage at the appropriate temperature. At each timepoint, the potency of each phage was assessed by EoP method in triplicates. *T*_0_ was measured before the initiation of storage.

### Spotting assay and EoP

For counting of phage titers, phage lysates or the equal volume mix of SNIPR001 CAPs were serially diluted tenfold in SM buffer or PBS, respectively. Bacterial lawns were prepared by adding 100 µl or 300 µl of bacterial overnight culture to 3 ml or 10 ml of 0.5% top agar (containing Ca^2+^ and Mg^2+^), which was vortexed briefly and poured onto a round or square LB plate. Five microliters of the dilution series of test phages were then spotted on lawns and left to dry at room temperature with an open lid before incubation at 37 °C overnight. The strains b52, b2479 and b17 were used as controls of the assay and included in each round of assays.

The next day, results were assessed (Extended Data Table 4). In this assay, a susceptible strain is defined as one producing plaques that are countable in PFU per milliliter as well as one without visible plaques but demonstrating impairment of bacterial growth (that is, lysis zones). Coverage defines the percentage of the total number of susceptible strains. Images of all plates were recorded. Figures illustrating EoP results first had titers log_10_ transformed and then standard deviances and averages were calculated subsequently. The clinical panels and control strains were tested in two independent experiments.

### Animals and housing

Mouse studies were performed with female CD-1 IGS mice (approximately 6–7 weeks of age upon arrival) from Charles River. The animals were housed in groups of three to five mice per cage within a climate-controlled room (temperature, 20–23 °C; relative humidity, 30–70%) under a 12 h light/12 h dark cycle (illuminated, 07:00–19:00). Standard pelleted chow and tap water were available ad libitum. Animals were allowed an acclimatization period of at least 7 d before the start of the experimental procedures. Thirty female Göttingen minipigs (approximately 4–7 months of age upon arrival) from Ellegaard Göttingen minipigs A/S were used for tolerability and kinetic studies. Animals were allowed an acclimatization period of at least 14 d before the start of experiments. Pigs were housed in groups of two to three animals and given standard pig diet twice daily and tap water was available ad libitum. All procedures were conducted in accordance with guidelines from the Danish Animal Experiments Inspectorate, Ministry of Environment and Food of Denmark and in accordance with the institutional license (BioAdvice, animal license 2015-15-0201-00540).

### Mouse gut colonization model

The mouse gut colonization model was adapted from ref. ^44^. Briefly, pretreatment with streptomycin (5 g l^−1^) in the drinking water was given 3 d before inoculation with *E. coli* b17 to decrease the level of native bacteria. On day 0, an inoculum of 3 × 10^7^ CFU of *E. coli* b17 was prepared from a frozen glycerol stock and administered to all mice in 0.25 ml by oral gavage.

Treatment was administered three times daily for 2 d starting 2 d after inoculation. Right before each administration, the four CAPs were mixed in a 1:1:1:1 ratio to form SNIPR001 at a high, medium or low concentration resulting in dose levels of 2 × 10^11^, 2 × 10^9^ and 1 × 10^7^ PFU. At the time of treatment, mice were administered 0.1 ml of 10% sodium bicarbonate by oral gavage followed by the oral administration of 0.3 ml of SNIPR001, saline (vehicle) or 43.5 mg kg^−1^ gentamicin.

### CAP recovery and tolerability studies

Göttingen minipigs were first given a cocktail of antibiotic comprising neomycin (60 mg kg^−1^, orally, once daily for 4 d) and cefquinome (2 mg kg^−1^, intramuscular once daily for 3 d) before SNIPR001 or single CAP administration to decrease the level of Gram-negative bacteria in the GI tract and therefore limiting phage replication. Animals were then fasted overnight and lightly sedated before administration of a single CAP, or SNIPR001 cocktail, once orally at 2 × 10^12^ PFU in 100 ml, following an oral administration of 50 ml of 10% sodium bicarbonate. Fecal samples were collected daily for CAPs quantification by plaque assay. In addition, for the tolerability study, blood samples were collected for hematology and blood chemistry analysis, including C-reactive protein, and plaque assay. Animals were closely monitored following SNIPR001 administration, and their body temperature was recorded regularly.

### Quantification of *E. coli* b17 and CAPs in feces

Fecal samples were homogenized and serially diluted in SM buffer. Triplicates of 10 μl of each dilution were then spotted on McConkey agar plates (Sigma-Aldrich, M7408) supplemented with streptomycin (1 mg ml^−1^) and incubated for 12–16 h at 37 °C for *E. coli* enumeration.

Plaque assays were performed for enumeration of CAPs in feces samples. Briefly, homogenized samples were centrifuged at 10,000*g* for 10 min, and the supernatant was serially diluted. Triplicates of 10 μl of each dilution were spotted on an *E. coli* b17 overlay and incubated for 12–16 h at 37 °C.

To quantify the presence of in vivo resistors, three colonies from each mouse fecal sample in the medium dose group at three different time points were picked from the McConkey agar plates. Colonies were incubated for 12–16 h at 37 °C in LB broth and used to make top agar overlays on LB agar plates. Then, plates were dried for 15 min in the LAF bench. The SNIPR001 cocktail, as well as the four individual CAPs, were spotted as a dilution series from 1 × 10^5^ PUF ml^−1^ stocks. As a control, a top agar overlay of colonization strain *E. coli* b17 was spotted in the same way. Plates were left to dry in the LAF bench with the lid on and subsequently incubated upside down for 12–16 h at 37 °C.

### Whole genome sequencing of *E. coli* strains from JMI

Total genomic DNA was extracted and purified using the KingFisher Cell and Tissue DNA kit (Thermo Fisher Scientific) in a robotic KingFisher Flex Magnetic Particle Processor (Thermo Fisher Scientific) workstation.

Total genomic DNA was used as input material for library construction. DNA libraries were prepared using the Nextera XT library construction protocol and index kit (Illumina) and sequenced on a MiSeq Sequencer (Illumina) using MiSeq Reagent Kits v3 (600 cycles).

### Resistance phenotype definitions

The extended-spectrum β-lactamase-phenotype was defined for *E. coli* as a minimum inhibitory concentration (MIC) value ≥2 mg l^−1^ for ceftriaxone, ceftazidime and/or aztreonam (https://clsi.org/).

Carbapenem-resistant *Enterobacterales* was defined as any isolate displaying imipenem, doripenem and/or meropenem resistance with MIC > 2 mg l^−1^ (https://clsi.org/).

### Assembly of whole-genome sequencing data

Raw sequencing reads were trimmed using Trimmomatic^57^ (version 0.39) with the settings ‘LEADING:3 TRAILING:3 SLIDINGWINDOW:4:15 MINLEN:36’. Trimmed reads were assembled using SPAdes^58^ (version 3.14.1) with default settings. Contigs shorter than 500 bp or with a sequencing depth below two times were removed from the final assemblies.

### Comparative genomic methods for clinical *E. coli* strains

MLST was performed using MLST2 (ref. ^59^) on the assembled genomes of the *E. coli* bacteria using default settings, with the MLST database downloaded on 1 July 2021, from the MLST2 repository (https://bitbucket.org/genomicepidemiology/mlst_db/src/master/). Phylogroup classification was conducted using ClermonTyping^60^ on the assembled *E. coli* genomes using default settings. Distance matrices for phylogenetic tree construction were generated using MASH^61^ with a k-mer size of 21 and 10,000 sketches per genome. Sketches were then compared to create the MASH distance in a pairwise manner to create a distance matrix of *E. coli* genomes.

### Phage synteny analysis

To generate the synteny plot, WT sequences of the four phages included in the final cocktail, plus the two closely related and well-known reference phages (RB69 AY303349.1 and T2 NC_054931.1) were annotated with RAST to extract predicted protein sequences. All protein sequences for each phage were queried again all other phage genomes using tblastn (v 2.12.0), with an *E*-value cutoff of 1 × 10^−10^. The synteny plot was then generated using a custom Python (v 3.7.10) script (see Data availability), using the drawSvg library (v 1.9.0). The plot shows the phage genomes in order of similarity and displays all tblastn hits as synteny blocks shaded by their protein identity. The proteins of the two reference phages were manually classified as belonging to each of the functional groups ‘DNA metabolism’, ‘structure’ or ‘other’ and colored accordingly.

### Data processing and visualization

Figures and key statistics were generated using R version 4.1.0. For figure generation, the following packages were used: RcolorBrewer v. 1.1-2, ape v. 5.5, ggsignif v. 0.6.2, ggpubr v. 0.4.0, matrixStats 0.59, reshape2 v. 1.4.4, ggimage v. 0.3.0, here v. 1.0.1, purr v. 0.3.4, ggtree^62^ v. 3.0.2, systemfonts v. 1.0.2, Cairo v. 1.5-12.2, cowplot v. 1.1.1, reaxxl v. 1.3.1, ggplot2 v.3.3.3, openxlsx, v. 4.2.3, patchwork v. 1.1.1, dplyr v. 1.0.7 and ggh4x v. 0.2.3. Averages and standard deviations are calculated after transforming the values to the scale shown on a given figure, for example when a log_10_ scale is used, the averages and standard deviations are calculated after log_10_ transformation.

### Reporting summary

Further information on research design is available in the Nature Portfolio Reporting Summary linked to this article.

## Online content

Any methods, additional references, Nature Portfolio reporting summaries, source data, extended data, supplementary information, acknowledgements, peer review information; details of author contributions and competing interests; and statements of data and code availability are available at 10.1038/s41587-023-01759-y.

### Supplementary information

Supplementary Information Supplementary Figs. 1–25 and Supplementary Tables 1–6.

Reporting Summary

## Data Availability

All data and results that were generated during this study are deposited at https://github.com/sniprbiome/SNIPR001_paper. Additional data are available in the Article, Online methods and Supplementary tables. To reproduce the results, no further data is needed. Phage genome sequences are deposited at Genbank under access numbers OQ067373 – 76. The MLST database was downloaded on July 1, 2021, from the MLST2 repository (https://bitbucket.org/genomicepidemiology/mlst_db/src/master/). For annotation of the CAP sequences, the following tools and datasets were used ResFinder 4.1 (https://cge.cbs.dtu.dk/services/ResFinder), VirulenceFinder-2.0 (https://cge.cbs.dtu.dk/services/VirulenceFinder/), PHASTER Prophage/Virus DB (https://phaster.ca/).

## Extended data

**Extended Data Table 1:** a Overview of the 15 CAPs generated from the selected WT phages resulting in four CAPs making up SNIPR001. The *E. coli* genes targeted by the five individual spacers and the sequence are listed below. b The *E. coli* genes targeted by the five individual spacers, that make up the array, and the sequence used in the CAPs

Backbone Phage	CAP	Arrays	Cas and Arrays	Tail fiber engineering (original location/added location)	Select based on marginal utility (Supplementary Fig. 7)	Select based on *in vivo* PK (Supplementary Fig. 8)	Select based on accelerated stability at 40 °C (Supplementary Fig. 9)	Select based on host range and *in vivo* efficacy (Supplementary Fig. 11)
15	α15.2	1-2-3	Separate sites	α15 wt/ α17	✓	✓	✓	✓
	α15.4	1-2-3	Separate sites	α17/ α21	✓	✓	×	
17	α17.2	4-5	co-transcribed	NA	✓	✓	×	
20	α20.4	4-5	co-transcribed	NA	✓	✓	✓	✓
31	α31.3	4-5	co-transcribed	NA	×			
	α31.4	4-5	co-transcribed	NA	×			
33	α33.3	4-5	co-transcribed	NA	×			
	α33.4	4-5	co-transcribed	NA	×			
46	α46.3	4-5	co-transcribed	NA	×			
	α46.4	4-5	co-transcribed	NA	✓	✓	✓	×
48	α48.3	4-5	co-transcribed	NA	×			
	α48.4	4-5	co-transcribed	NA	✓	✓	✓	✓
51	α51.4	4-5	co-transcribed	NA	×			
	α51.5	4-5	co-transcribed	NA	✓	✓	✓	✓
	α51.6	4-5	co-transcribed	NA	✓	✓	✓	×
Total	15				8/15	8/8	6/8	4/6

spacer	Target gene	Sequence
1	*bolA*	AGTGGGAAGGGTTGCAGGACACCGTCTTTGCC
2	*rpoH*	CCGATGTTACCTTCCTGAATCAAATCCGCCTG
3	*fimH*	CGAATGACCAGGCATTTACCGACCAGCCCATC
4	*lptA*	TGATTGACGGCTACGGTAAACCGGCAACGTTC
5	*murA*	GCTGTTAACGTACGTACCGCGCCGCATCCGGC

**Extended Data Table 2:** List of databases used for the analysis of SNIPR001 CAP sequences

Database	Source	Analysis
ResFinder 4.1	https://cge.cbs.dtu.dk/services/ResFinder	Identification of acquired genes and/or chromosomal mutations mediating antimicrobial resistance
VirulenceFinder-2.0	https://cge.cbs.dtu.dk/services/VirulenceFinder/	Detection of virulence genes including exotoxins
PHASTER Prophage/Virus DB	https://phaster.ca/	Detection of potential transposases and integrases

**Extended Data Table 3:** Panel of bacterial strains (Aerobic: n = 6, Anaerobic: n = 3, Aerobic/Anaerobic: n = 1) tested via a biopotency assay, showing Gram-type classification, growth conditions and source/ID

Strain/name	Gram type	Growth condition	Source/ID
*Acinetobacter baylyi*	Negative	Aerobic	ATCC 33304
*Klebsiella pneumonia*	Negative	Aerobic	SNIPR Biome ID b2951
*Enterococcus faecalis* (Andrewes and Horder) Schleifer and Kipper-Balz	Positive	Aerobic	ATCC 47077
*Streptococcus thermophilus* Orla-Jensen	Positive	Aerobic	ATCC 19258
*Bacillus coagulans* Hammer	Positive	Aerobic	ATCC 7050
*Staphylococcus aureus* subsp. *aureus* Rosenbach	Positive	Aerobic	ATCC 12600
*Eubacterium limosum* Eggerth	Positive	Anaerobic	ATCC 8486
*Bacteroides vulgatus* Eggerth and Gagnon	Negative	Anaerobic	ATCC 8482
*Bacteroides thetaiotaomicron* (Distaso) Castellani and Chalmers	Negative	Anaerobic	ATCC 29148
*E. coli*	Negative	Aerobic/Anaerobic	Takara Cat. #636763

**Extended Data Table 4:** Criteria used to evaluate results of the spot assay and define strain susceptibility following standards^46,63^

Spot assay categories	Observation	Strain susceptibility to SNIPR001
Plaques	Visible plaques were counted, and PFU/mL was calculated by multiplying with volume and dilution	Susceptible strain
Lysis zone	Impairment of bacterial growth observed as lysis zones. No plaques are visible; the highest dilution of visible zones is recorded	Susceptible strain
Negative	Neither plaques nor lysis zones are detected	Non-susceptible strain

**Extended Data Table 5:** Exact P-values resulting from the statistical analysis of the data shown in Fig. 6D

Test used	Time point	Comparison group	Nominal P-value	FDR corrected with Holm’s method for timepoint
Two-sided Mann-Whitney U test	Day 2–8 hours after first dose. 1 dose total	SNIPR001 High	1,08E-05	3,25E-05
		SNIPR001 Medium	1,15E-02	2,30E-02
		SNIPR001 Low	1,26E-02	2,30E-02
	Day 3–24 hours after first dose. 3 doses total	SNIPR001 High	1,08E-05	3,25E-05
		SNIPR001 Medium	3,25E-04	3,25E-04
		SNIPR001 Low	2,17E-05	4,33E-05
	Day 4–48 hours after first dose. 6 doses total	SNIPR001 High	4,37E-04	1,31E-03
		SNIPR001 Medium	1,23E-01	1,23E-01
		SNIPR001 Low	4,87E-04	1,31E-03
Two-sided Kruskill-Wallis test	Day 2–8 hours after first dose. 1 dose total	SNIPR001 low, medium, and high	3,07E-03	NA
	Day 3–24 hours after first dose. 3 doses total	SNIPR001 low, medium, and high	2,07E-03	NA
	Day 4–48 hours after first dose. 6 doses total	SNIPR001 low, medium, and high	1,04E-02	NA

## Code availability

All code needed to produce this study is available at https://github.com/sniprbiome/SNIPR001_paper.
