# Supplementary material for: Engineered phage with antibacterial CRISPR–Cas selectively reduce E. coli burden in mice
Source: Nat Biotechnol. 2023 May 4;42(2):265–74. doi: 10.1038/s41587-023-01759-y (PMC10869271; doi:10.1038/s41587-023-01759-y)
Supplement: Supplementary file 1 — Supplementary Figs. 1–25 and Supplementary Tables 1–6. [file 41587_2023_1759_MOESM1_ESM.pdf]

nature biotechnology

Article https://doi.org/10.1038/s41587-023-01759-y

Engineered phage with antibacterial CRISPR–Cas selectively reduce *E. coli* burden in mice

In the format provided by the authors and unedited

SUPPLEMENTARY MATERIAL

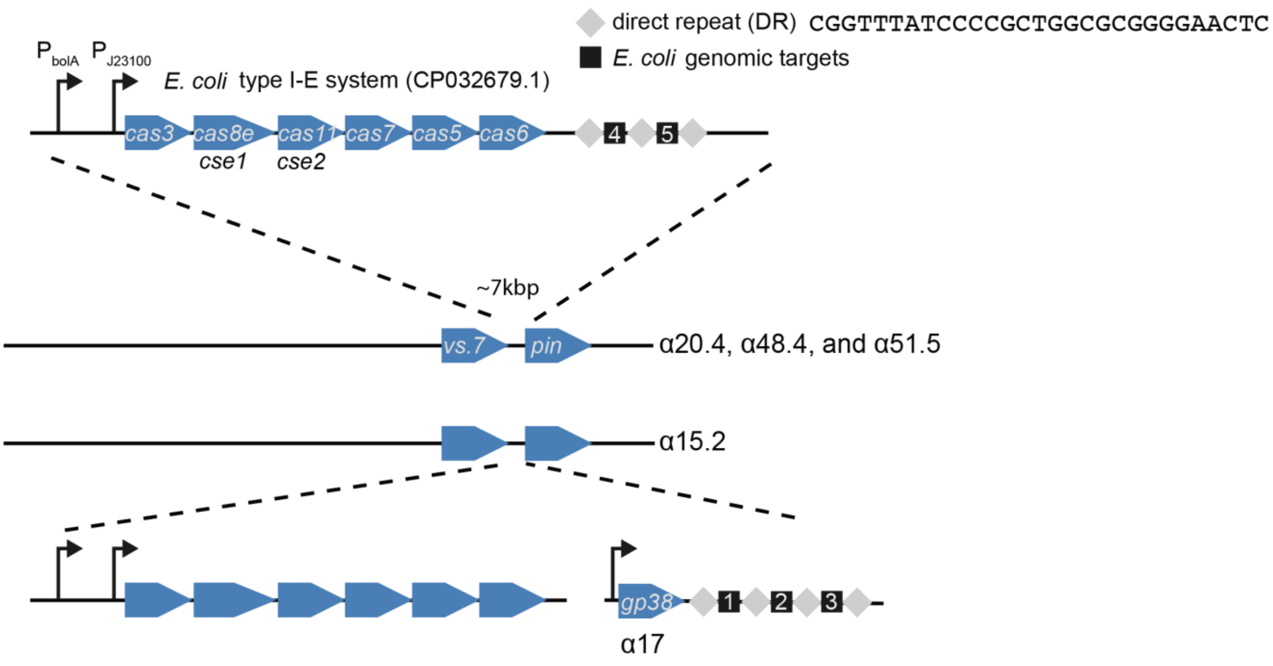

***Figure S1**: Overview of engineering using recombination of the CRISPR-Cas system into CAPS α20.4, α48.4, and α51.5 and addition of tailfiber gp38 from α17 into α15.2 (see material and methods). The type I-E CRISPR-Cas system from* E. coli *MG1655 (Genbank CP032679.1) was used in combination with matching direct repeat sequences CGGTTTATCCCCGCTGGCGCGGGGAACTC. The system is driven by the* P_{bolA} *promoter*[1] *with sequence (AACCTAAATATTTGTTGTTAAGCTGCAATGGAAACGGTAAAAGCGGCTAGTATTTAAAGGGATGGATGAC ATCTCAGCGTTGTCG) and* P_{J23100} *(TTGACGGCTAGCTCAGTCCTAGGTACAGTGCTAGC). The proteins encoded by* cas8e, cas11, cas7, cas5 *and* cas6 *make up Cascade (CRISPR-associated complex for antiviral defense) as shown in Figure 1. The blank boxes indicate the five different spacer sequences targeting the* E. coli *genome (spacer 1-3 in α15.2 and spacer 4-5 in α20.4, α48.4, and α51.5, listed in Extended Data Table 1b). The payload is inserted in the region between the* vs.7 *gene and the* pin *gene using recombination by homology resulting into CAPs α15.2, α20.4, α48.4, and α51.5.*

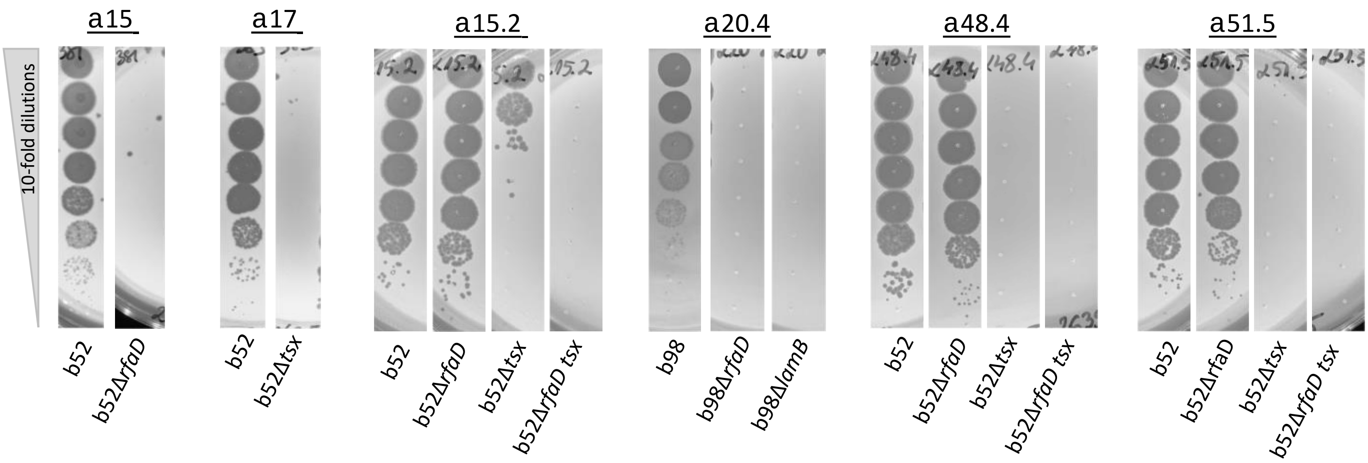

***Figure S2**: Efficiency of plating analyses of relevant phages on receptor knock-out mutants.* E. coli *strains b52 and b98 are broadly susceptible to tested phages in the collection. Therefore, using the standard Lambda red-based homologous recombination system*[2]*, an array of known phage receptors*[3] *was knocked out via insertion*

of a PCR-amplified selection marker (zeocin [zeoR, Sh ble] and/or spectinomycin resistance [spcR, aadA]) with homologous arms to respective genome positions. Surface proteins evaluated were: Tsx, OmpF, OmpA, OmpC, LamB, TolC, BtuB, FhuA, PhoE and FadL (Table S6). Transformants were selected on corresponding antibiotics, confirmed by PCR and Sanger sequencing, and subjected to efficiency of plating analyses. The representative images shown correspond to at least three biological replicates.

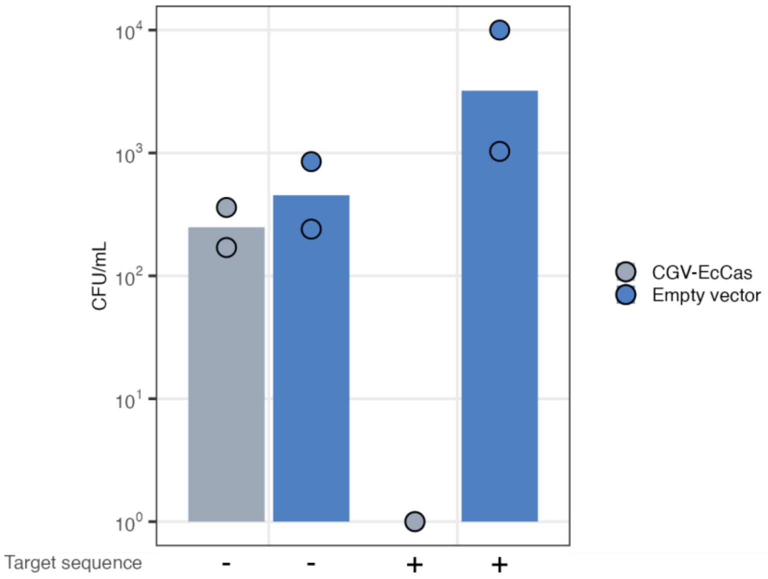

Figure S3*: Specific killing of target* E. coli *strain by CGV-EcCas. The killing efficiency of the CGV was assayed using* E. coli *target and non-target strains as recipients. Rate of conjugative transfer to the non-target recipient cells was similar for CGV-EcCas (blue) and for the control plasmid (grey). Cell viability is measured in CFU/mL. The CRISPR-Cas plasmid reduced the number of viable cells in the target strain by more than 3 log_{10} CFU/mL while not effecting the growth of non-target strain. Points indicate individual replicates, and bars indicate averages.*

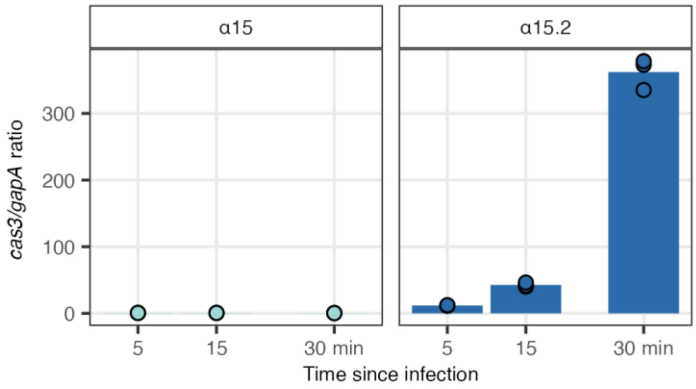

Figure S4: *RT-qPCR showing ascending relative abundance of* cas3 *transcripts over time in a synchronized infection of* E. coli *with WT α15 and CAP α15.2 at MOI of 1.* Cas3 *transcripts were only detected upon CAP α15.2 infection. Cas3 expression was measured relative to household gene* gapA. *Bars indicate average value between three biological replicates, while points indicate individual replicates, and bars indicate averages.*

-

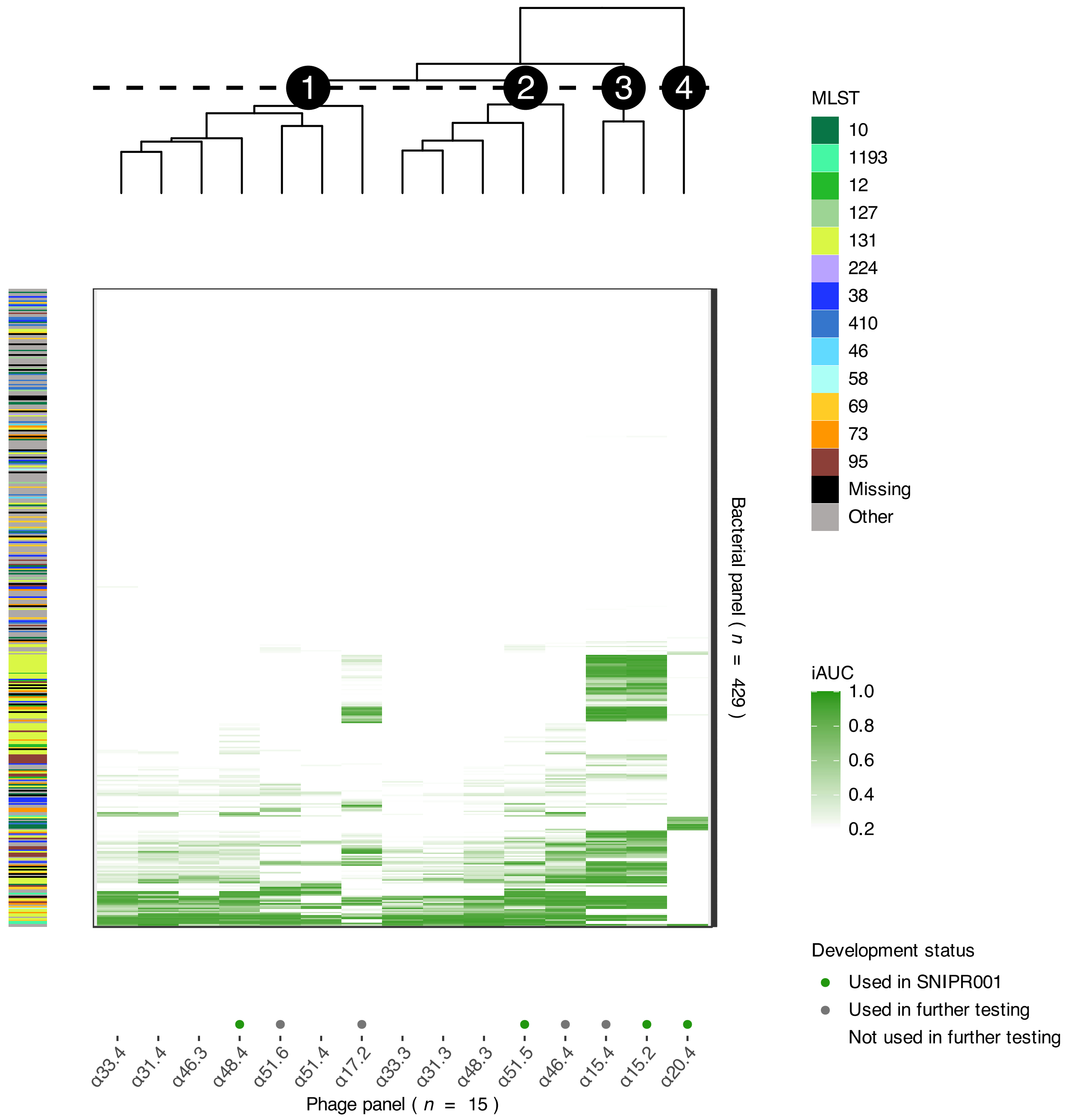

***Figure S5**: Secondary screening of 15 CAPs against panel of 429* E. coli *strains resulting in four distinct host range clusters. Host range profiles are clustered and used to separate the CAPs in four distinct clusters. Subsequently 8 CAPs being (SNIPR001 components highlighted in green, others later deselected in gray) evaluated for PK and efficacy* in vivo.

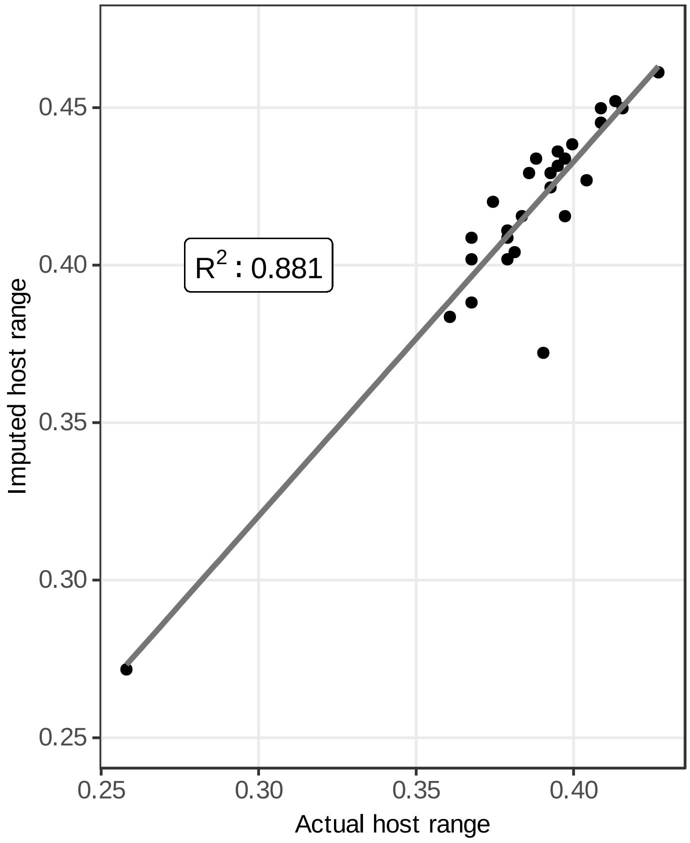

Figure S6: *Imputed host ranges of CAP combinations (horizontal axis) and actual host ranges of those CAP combinations (vertical axis). Combinations of 3 CAPs, as well as the CAPs composing those combinations were screened against a representative* E. coli *panel (*n = *429) A bacterial strain was considered inhibited if iAUC>0.2. Imputed host range was calculated in a complementary way, i.e. if one or more of the composing CAPs had an inhibitory effect the combination of those CAPs was predicted to also have an inhibitory effect.* R^2 *was obtained from a linear model using functions lm and summary in R.*

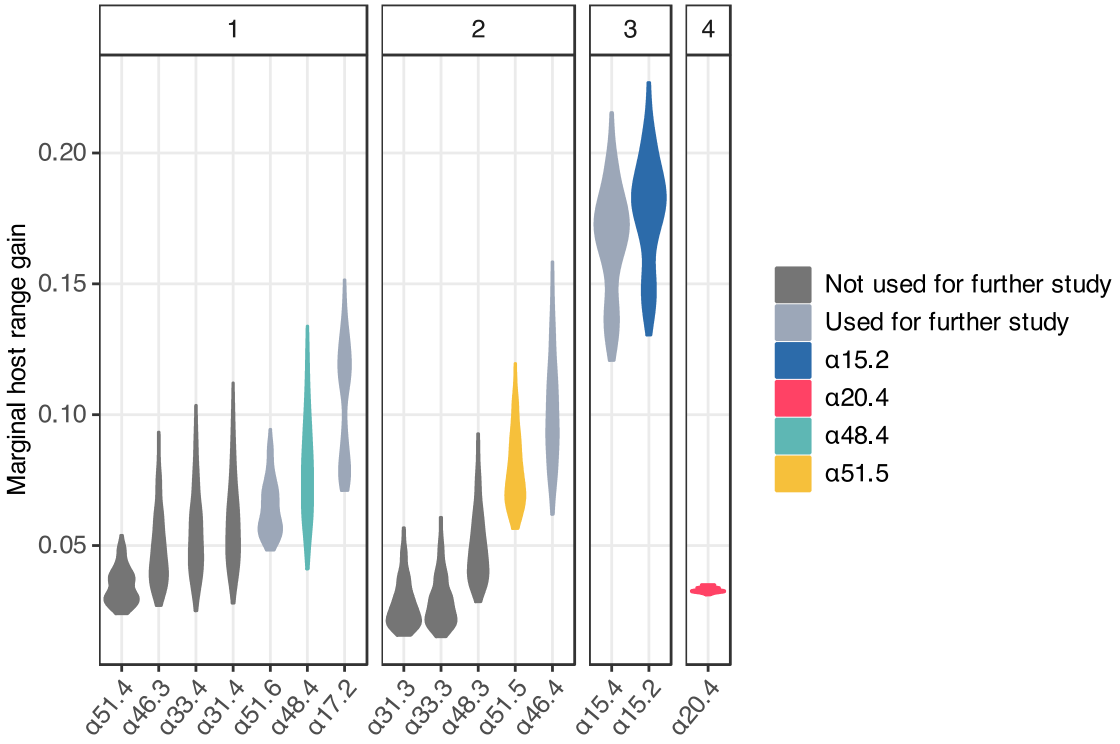

Figure S7*: Simulated marginal host range gain of each of the 15 evaluated CAPs from Fig S5*. In silico *cocktails of 3 components were constructed and the contribution of the fourth CAP was calculated, the*

distribution is shown for each individual CAP. In silico *cocktails that resulted in two or more phages originating from the same wild-type phage were excluded, due to a large overlap and thus a poor, unrepresentative performance. The CAPs are grouped by their host range cluster from Fig S5 (top header). The lowest seven ranking phages were deselected (dark gray) based on their limited contribution to the host range of a four-component cocktail.*

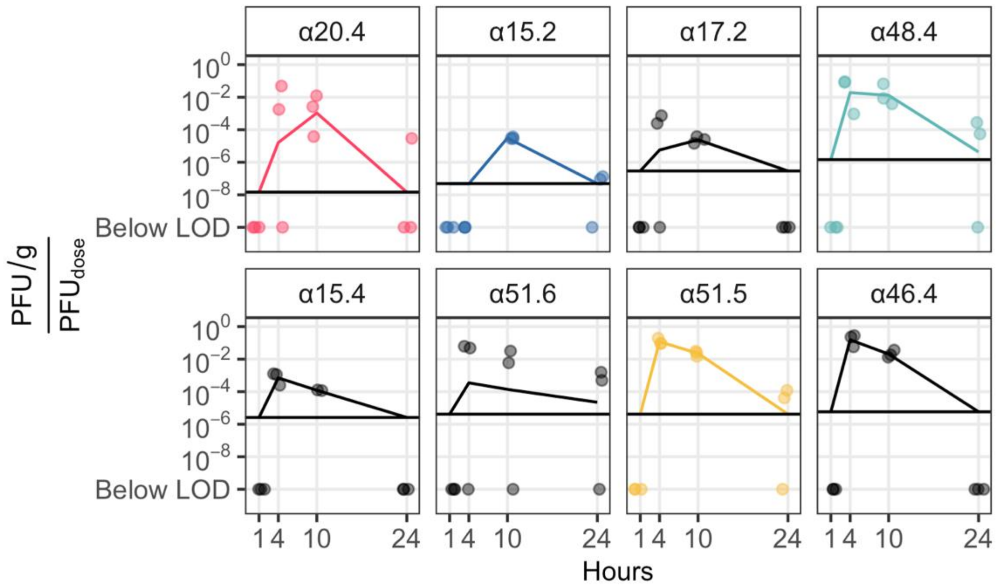

Figure S8: As part of the selection process eight CAPs were assessed in a mouse PK model to establish the recovery in feces (PFU/g) normalized by the phage dose given at t_0. The CAPS that are part of SNIPR001 are highlighted by their cognate color. All CAPs could be recovered in the fecal samples.

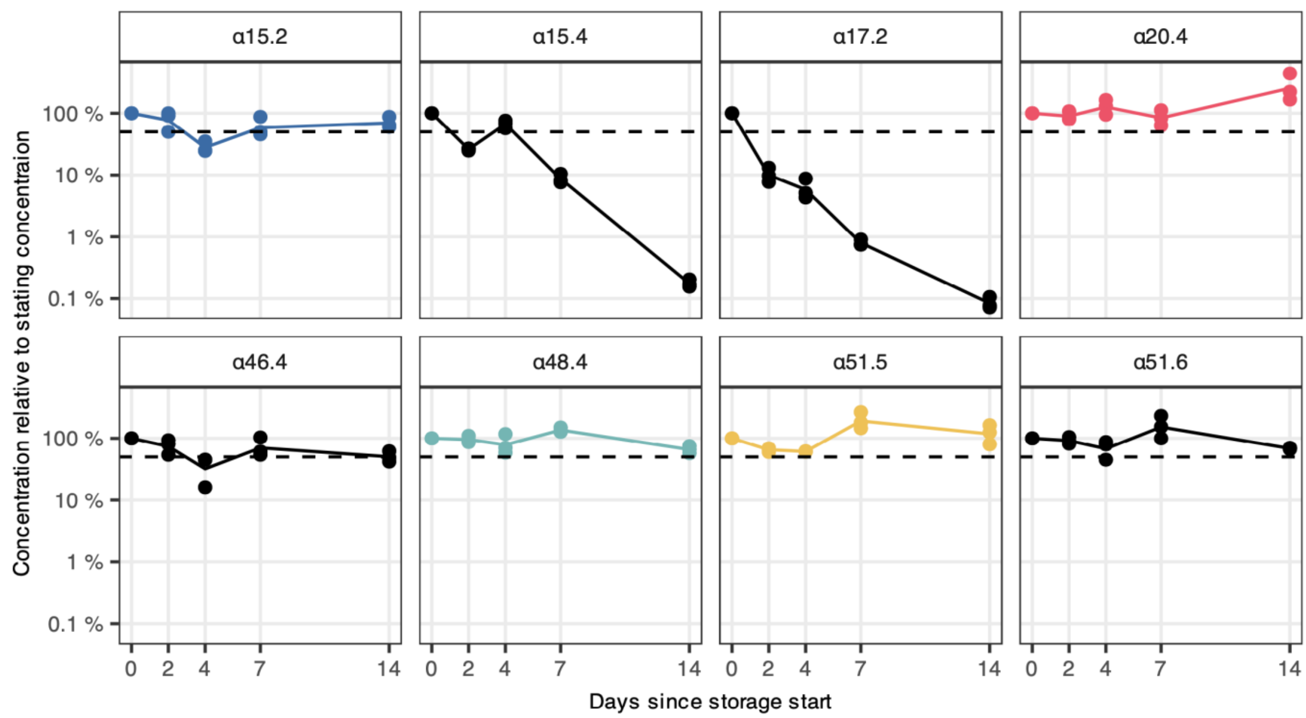

Figure S9: *Individual CAPs* (n = 3) *were stored at 40 °C for 14 days and the titer was assayed at five timepoints. The normalized titer (compared to t_0) shows a decrease to below 1% for α15.4 and α17.2, which were subsequently deselected for further evaluation. The CAPs making up SNIPR001 are highlighted in color.*

Dashed line indicates 50% of initial potency. Lines indicate mean stating concentration while individual points indicate replicates.

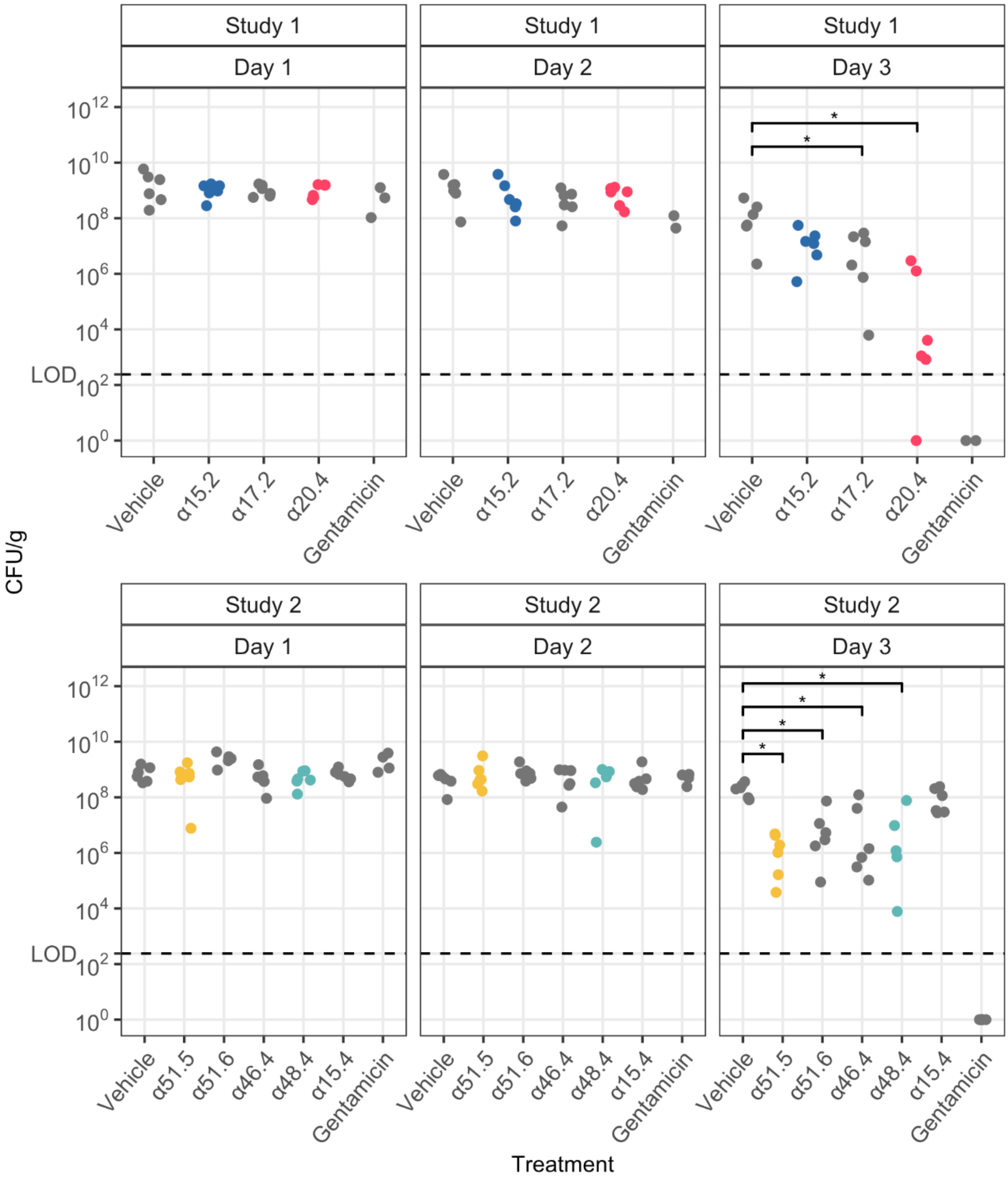

Figure S10: *Efficacy measurement of the individual CAPs using the murine* in vivo *model. The efficacy was measured by* E. coli *b17 recovery after oral administration of eight CAPs* (n = 6)*, and gentamicin* (n = 3)*. Statistical analyses were performed using an unpaired Mann Whitney U test and FDR corrected using Holm's method. P-values are adjusted using Holm's method all comparisons are made to vehicle treated cohort. P-values for study 1, day 3: 0.03, 0.013, for α15.2 and α17.2, respectively. P-values for study 2, day 3: 0.011, 0.011, 0.017, 0.013, for α51.5, α51.6, α46.4, and α48.4, respectively .*

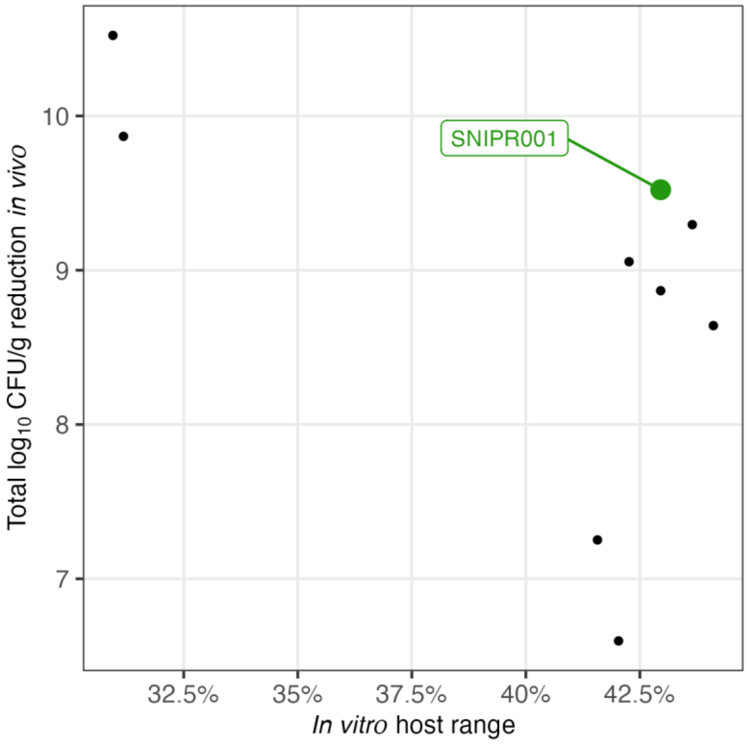

Figure S11: *Predictions of* in vitro *host range based complimentary OmniLog screenings (horizontal axis) and calculated total* in vivo E. coli *(CFU/g) reductions compared to vehicle administration in mice after 3 days (vertical axis), for combinations (*n *=9) of 4 CAPs, excluding combinations with multiple CAPs that originate from the same WT phage. The selected SNIPR001 combination α15.2 α20.4 α48.4 and α51.5 is highlighted in green.*

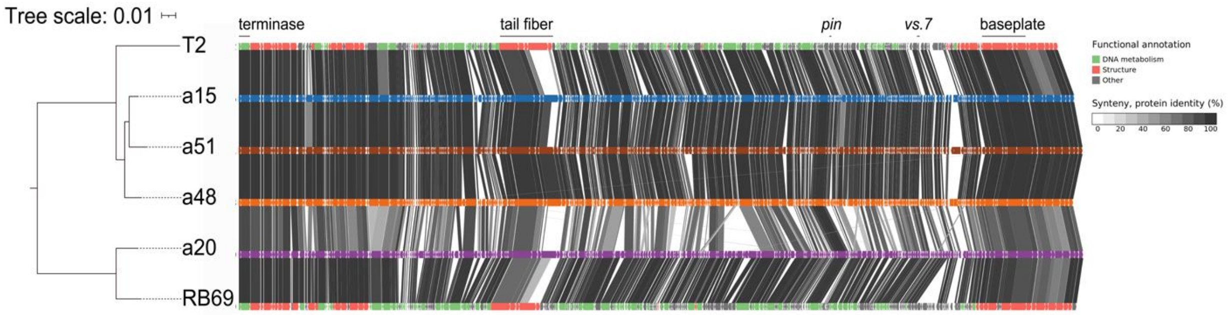

Figure S12: Genomic overview of wild type phages α15, α48, and α51 and α20, with on top the T2 reference genome (Genbank ID NC_054931.1) and bottom RB69 reference genome (GB ID: AY303349.1). Protein synteny is shown with gray blocks between the genomes, where the brightness represents the protein similarity ranging from 100% to 0%. Functional groups are annotated by green and red coloring of the genes, the CRISPR-Cas constructs were engineered in the region between pin *and* vs.7 *as described in Figure S1.*

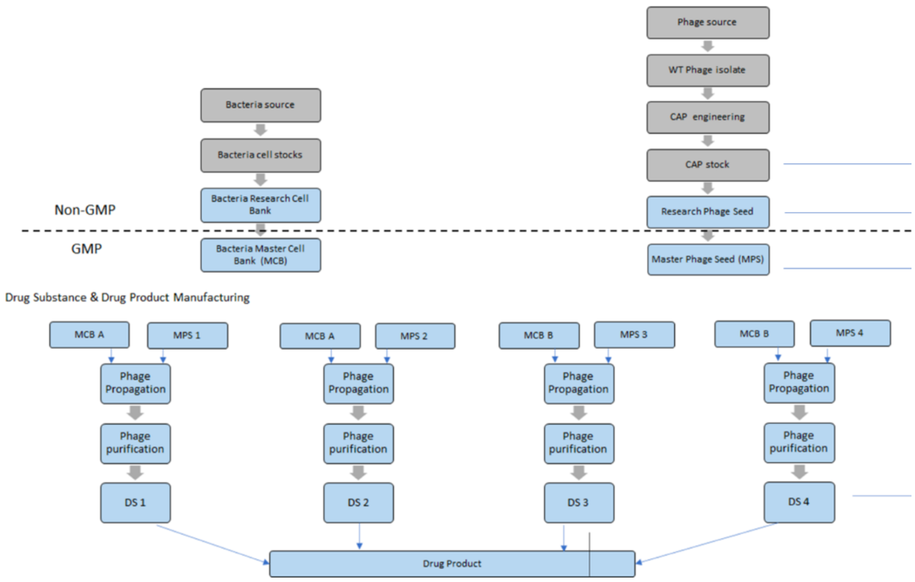

Figure S13: *Flowchart of the non-GMP and GMP processes to develop a CRISPR-Cas-armed page drug product starting with a bacterial source and phage source. The research process is shown in gray and the production process in blue.*

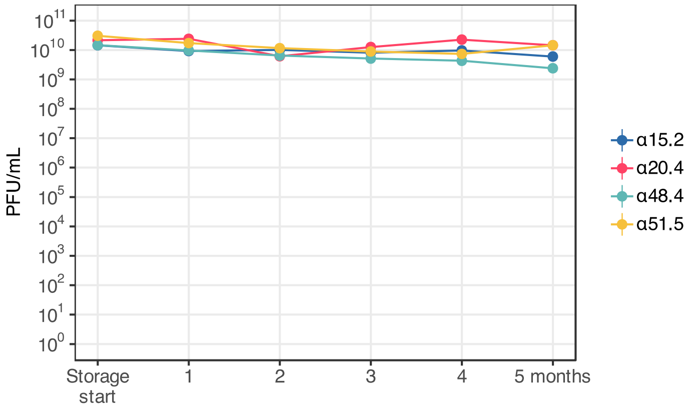

Figure S14: The potency of the individual components of SNIPR001 is not impacted as measured by titers based on plaque forming units (PFU/mL) over 5 months of 2–8°C storage in upright containers shown as an average of triplicate experiments, which are indicated by individual dots, and line indicates averages.

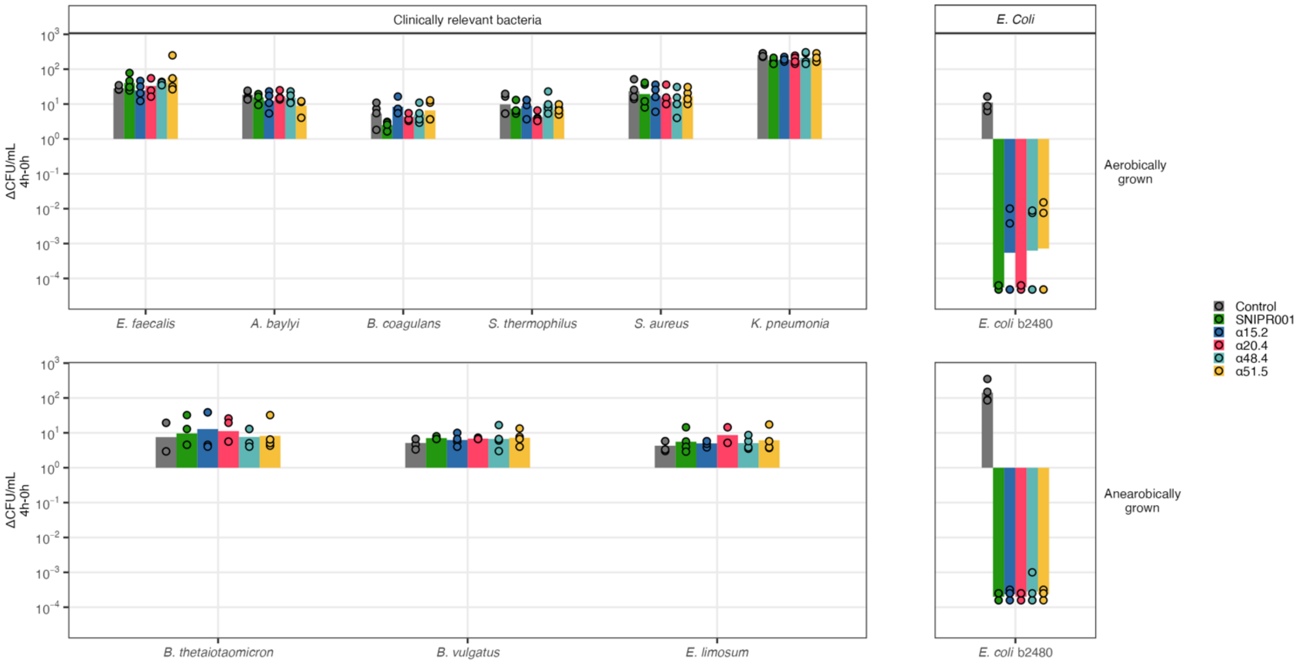

Figure S15*: Specificity evaluation of SNIPR001 and individual CAPs against a panel of clinically relevant bacteria and* E. coli *strain b2480 (positive control) showing no off-target effects. Positive values represent bacterial growth (absence of observed killing), while negative values indicate bacterial killing following phage treatment within the assessed time-period. All values indicated as means of four biological replicates with points indicating replicates, and bars indicate averages, measuring growth over a 4-hour period, measured in CFU/mL.*

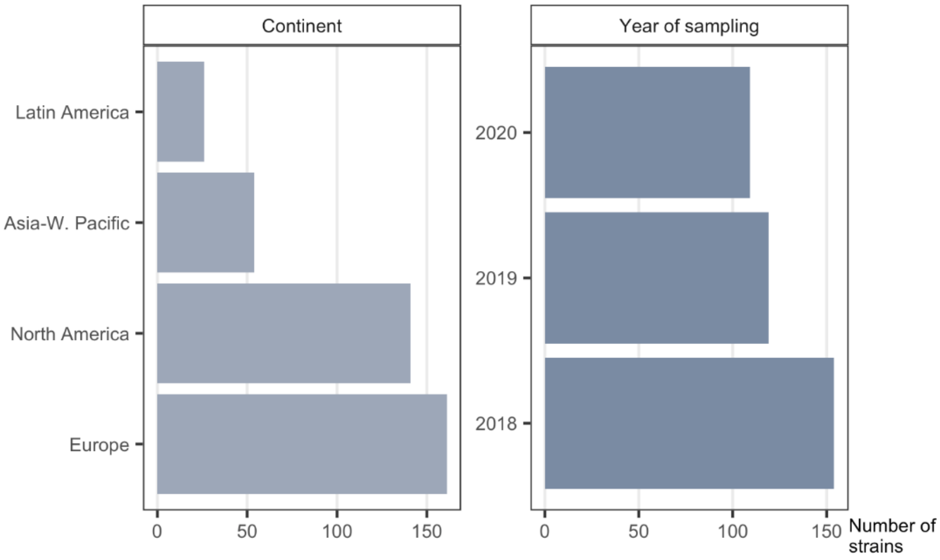

Figure S16: *The 382* E. coli *clinical strain panel collected by JMI is recent (right) and of broad geographic coverage (left).*

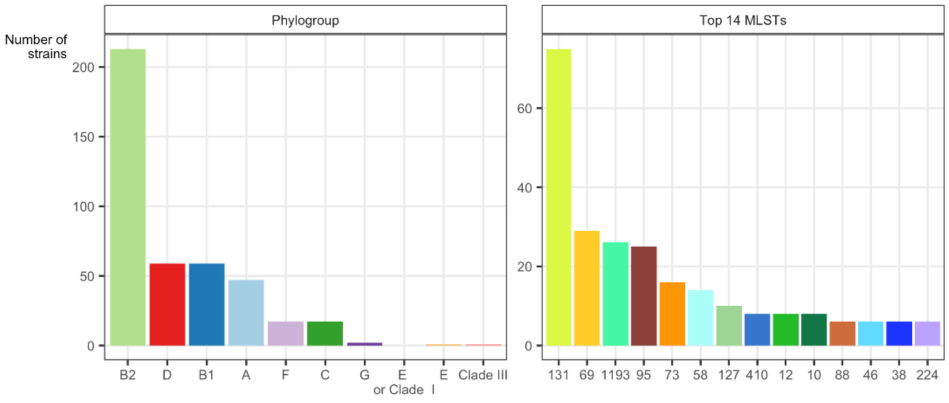

Figure S17: *Clinical* E. coli *strain panel (*n *= 382) has a diverse distribution as characterized by both phylogroup typing (left) and MLST (right).*

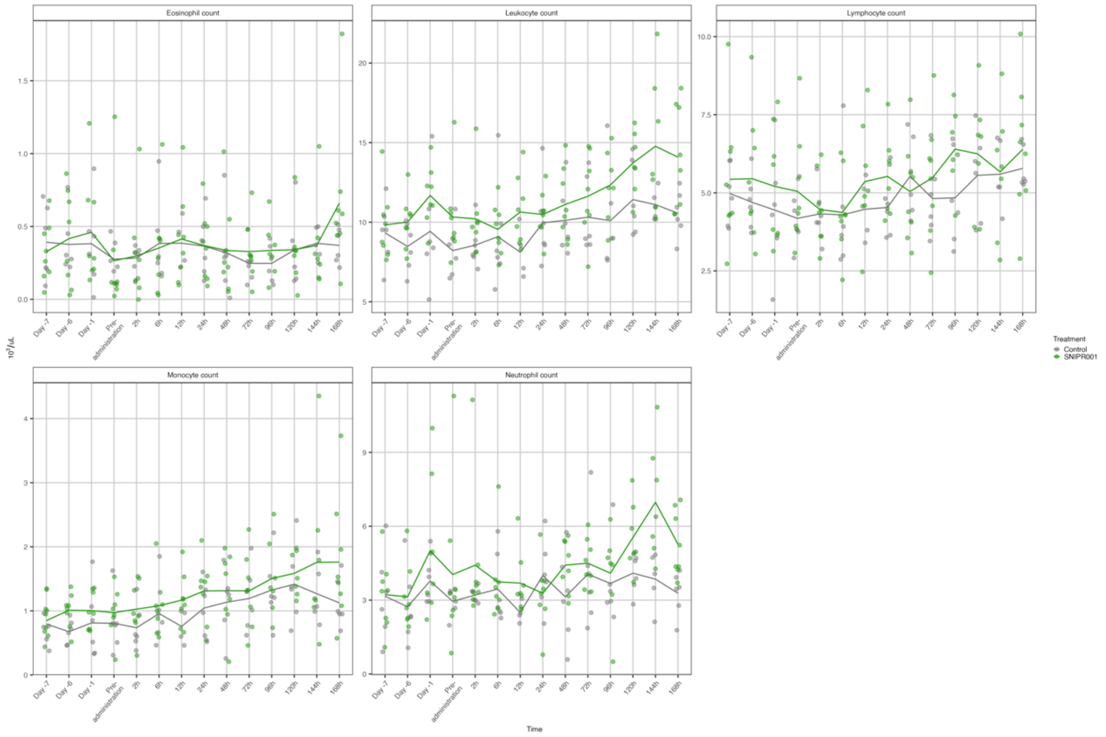

Figure S18: *Hematology parameters (monocytes, neutrophils, eosinophils, lymphocytes and leukocytes) measured in minipigs over the course of the study. The results showed no difference between the SNIPR001- and vehicle-treated pigs (grey and green, respectively) as lines indicating the average cell count (*n *= 6 for vehicle,* n *= 8 for SNIPR001, with duplicate samples for pre-administration), and points indicating individual observations, with lines indicating averages.*

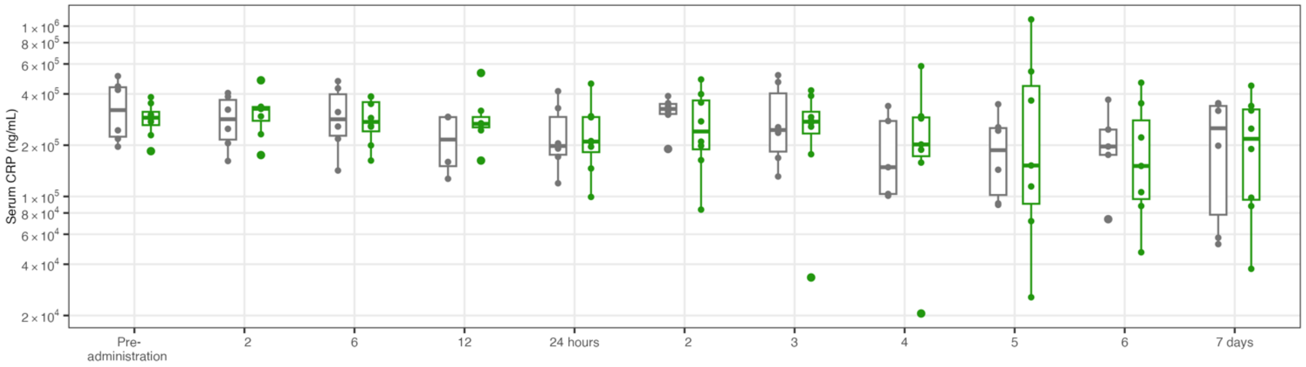

Figure S19: *Serum C-reactive protein measurements (ng/mL) in minipigs. Data are presented as boxplots with all individual measurements included as points (*n *= 6 and* n *= 8 for vehicle and SNIPR001, respectively). Whiskers of the boxplot illustrate maximum and minimum points, the upper and lower bounds illustrate the 75*th *and 25*th *percentiles, respectively, and center line indicates the distribution median. The results showed no difference between the SNIPR001- and vehicle-treated pigs (grey and green, respectively).*

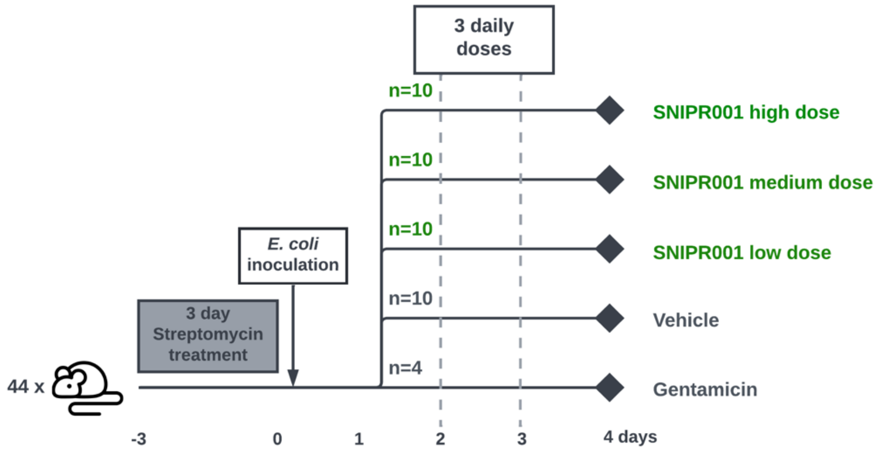

Figure S20: *Schematic overview of the murine efficacy model used to test SNIPR001 in Fig 6C-D.*

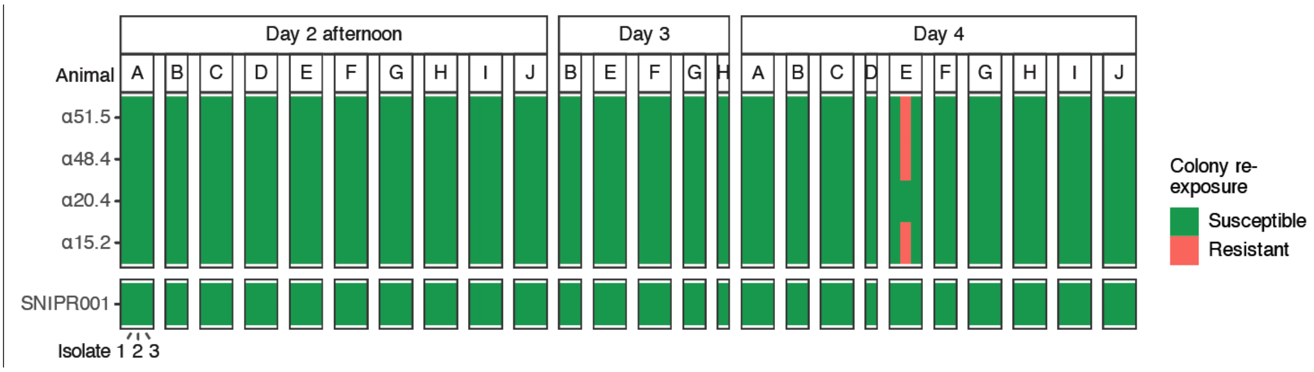

Figure S21: *Each clone (*n = 3*) isolated from mice (*n = 10*) feces at day 2, day 3 and day 4 was challenged with the SNIPR001 cocktail and the four individual phages comprising the cocktail. Phage susceptibility was assessed as a comparison to the colonization strain used in the efficacy studies where less phage susceptibility relates to resistance to the phage. The results indicate that 66 of the recovered isolates showed no resistance to the phage cocktail by plaque assay, only on the last day one colony was found resistant to α51.1, α48.4 and α15.2. Twenty-four of the 90 isolates could not be regrown in liquid culture due to residual presence of phage.*

Overview of all parameters measured (listed in **Table S5**) during the dosing of all four CAPs (α15.2, α20.4, α48.4 and α51.5) in Gottingen minipigs over the course of seven days during the experiment shown in **Fig. 6A** of the main text.

The measured electrolytes are shown in **Fig. S22**, the hepatic parameters in **Fig.S23**, the renal parameters in **Fig. S24** and the hematological parameters in **Fig. S25**. The minipigs exhibited no clinical signs and no significant changes were observed in hematology or biochemistry parameters compared to vehicle treatment, supporting that SNIPR001 was well tolerated.

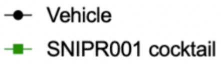

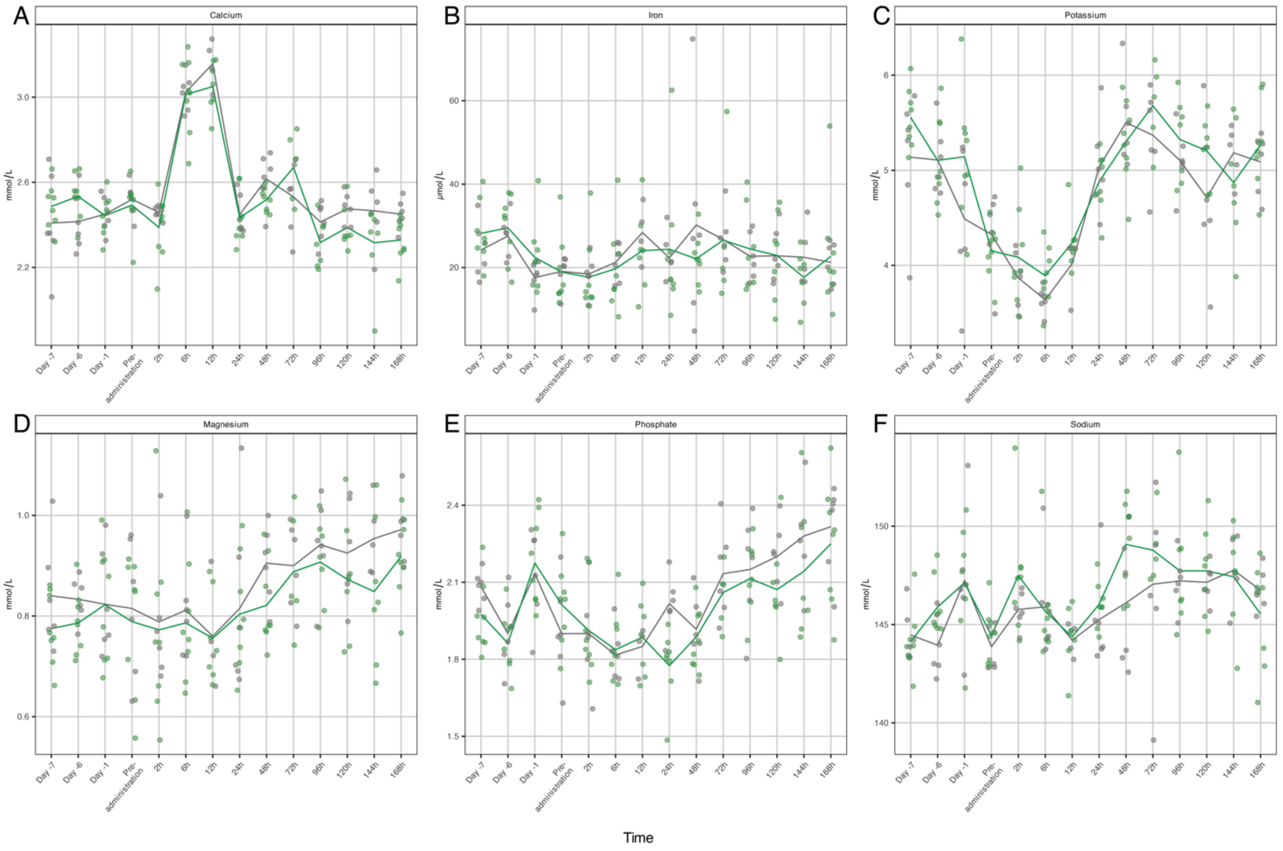

Figure S22: *Electrolytes a) calcium, b) iron, c) potassium, d) magnesium, e) phosphate and f) sodium were determined by taking blood samples for serum of the Gottingen minipigs. Collection was performed without use of clot activator. Data illustrated as lines for average values, and dots for replicates, for groups administered SNIPR001 (*n *= 8, with duplicates for pre-administration) shown in green, and vehicle (*n *= 6, with duplicates for pre-administration) shown in grey.*

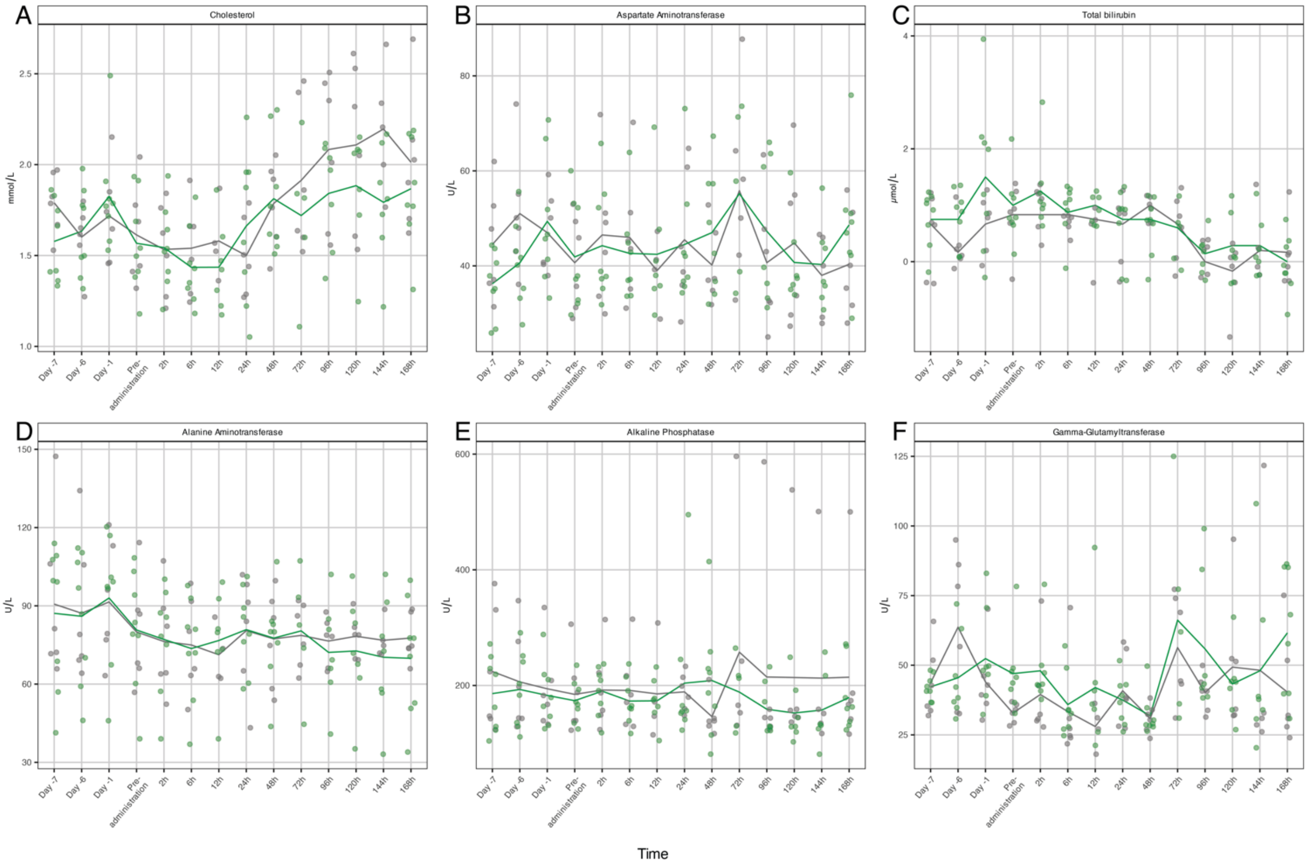

Figure S23: *Hepatic parameters a) cholesterol, b) aspartate aminotransferase, c) total bilirubin, d) alanine aminotransferase, e) alkaline phosphatase and f) gamma-glutamyl transferase, were determined by taking blood samples for serum of the Gottingen minipigs. Collection was performed without use of clot activator. Data illustrated as lines for average values for groups and dots for replicates administered SNIPR001 (*n = *8, with duplicates for pre-administration) shown in green, and vehicle (*n = *6, with duplicates for pre-administration) shown in grey.*

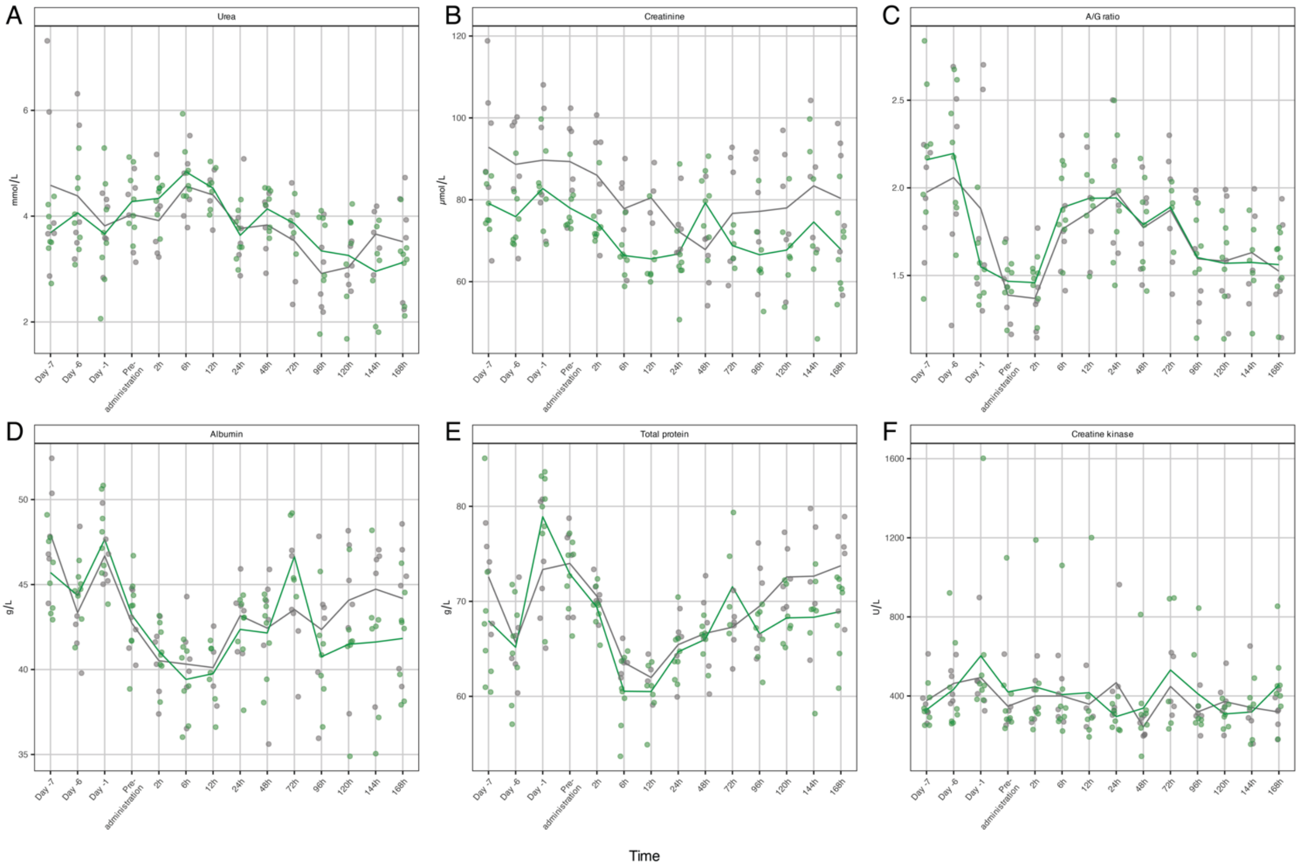

Figure S24: *The renal parameters a) urea, b) creatinine, c) albumin/globulin (A/G) ratio d) albumin, e) total protein and f) creatine kinase were determined by taking blood samples for serum of the Gottingen minipigs. Collection was performed without use of clot activator. Data illustrated as lines for average values and dots for replicates for groups administered SNIPR001 (*n *= 8, with duplicates for pre-administration) shown in green, and vehicle (*n *= 6, with duplicates for pre-administration) shown in grey.*

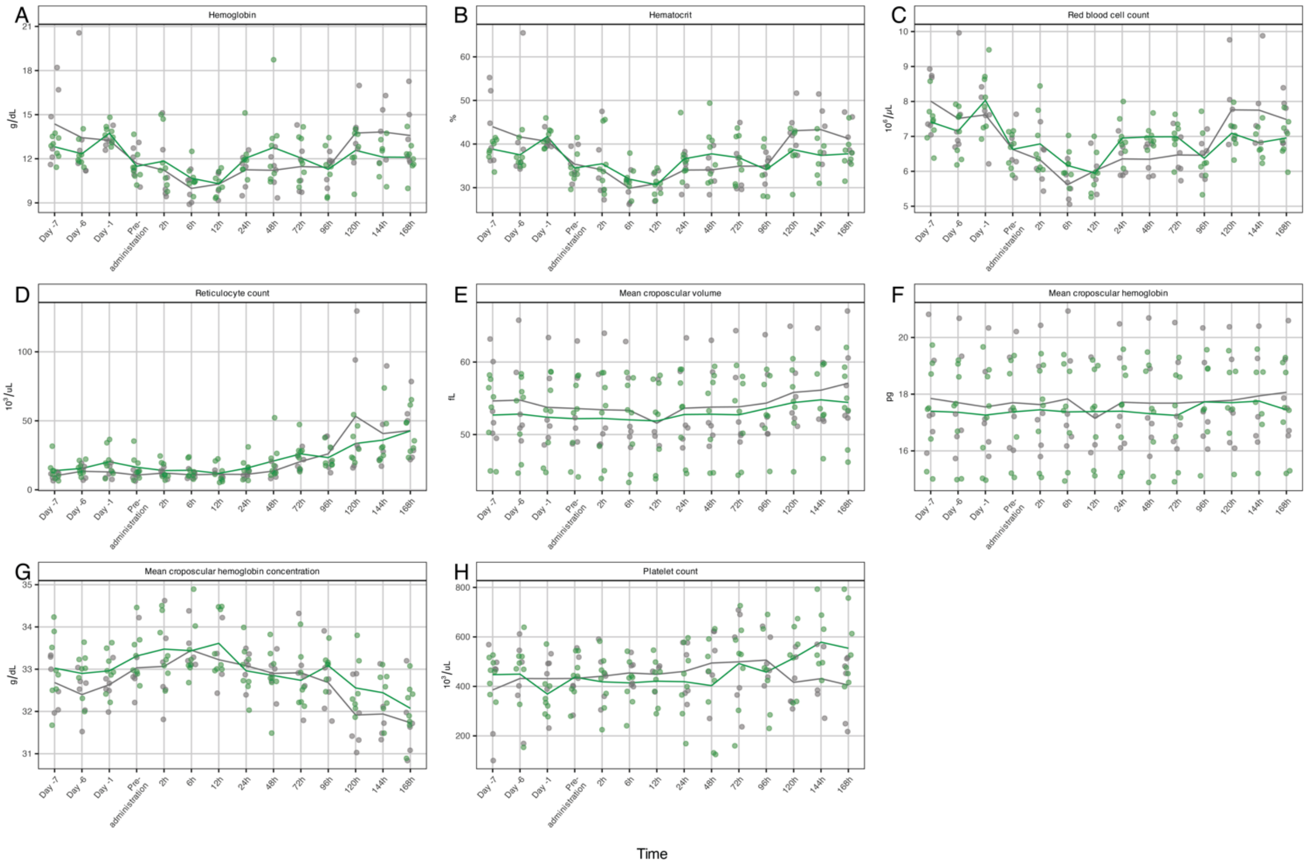

Figure S25: *Hematological parameters a) hemoglobin, b) hematocrit, c) RBC count, d) reticulocyte count, e) mean corpuscular volume, f) mean corpuscular hemoglobin, g) mean corpuscular hemoglobin concentration, h) platelet count of the Gottingen minipigs. White blood cell differentials were determined by analysis of full blood stabilized in K_3EDTA. Analysis was performed using a ProCyte Dx Hematology Analyzer (IDEXX) with minipig default settings. Data illustrated as lines for average values and dots for replicates for groups administered SNIPR001 (*n *= 8, with duplicates for pre-administration) shown in green, and vehicle (*n *= 6, with duplicates for pre-administration) shown in grey.*

Table S1: *Source and number of phages included in the screening panel (total* n *= 162)*

**Phage source**	*n*
isolated by SNIPR Biome from commercial and environmental sources	82
phage bank LyseNTech (Korea)	71
ATCC	2
University of Copenhagen	1
Kirikkale University (Turkey)	6
**Total**	162

Table S2: *Test criteria of the genetic features of the Master Phage Seed (MPS) and Drug Substance (DS) and the results for all four CAPs α15.2 α20.4 α48.4 and α51.5*

**Criteria**		**Master Phage Seed**	**Drug Substance**
**Nucleotide sequence level**			
	CRISPR-Cas	No mutations in CRISPR-Cas operon at >10% frequency	Consensus call (= >50%) is similar 100% on protein level of the CRISPR-Cas operon and the consensus call of the protospacers are 100% similar on nucleotide level.

	Intact host derived prophages	absent	absent
	Overall sequence	Consensus call (= >50%) is 99% similar	Consensus call (= >50%) is 99% similar
**Annotation level**			
	Lysogeny	absent	absent
	Antimicrobial resistance	absent	absent
	Toxins	absent	absent
	Virulence genes	absent	absent

Table S3: *Generalized transduction assay comparing positive control T4GT7 against negative control (T4 wild type) and CAPs α15.2, α20.4, α48.4, and α51.5. *ND = no transductants were detected*

<table>
<tr><th colspan="3">Positive control (T4GT7)</th><th colspan="3">Negative control (T4 wild type)</th><th colspan="3">CAP α15.2 DS; α20.4 DS; α48.4 DS; α51.5 DS</th></tr>
<tr><td colspan="9">Frequency of transduction</td></tr>
<tr><td>MOI 0.5</td><td>MOI 0.1</td><td>MOI 0.01</td><td>MO I 0.5</td><td>MOI 0.1</td><td>MOI 0.01</td><td>MOI 0.5</td><td>MOI 0.1</td><td>MOI 0.01</td></tr>
<tr><td>9.80 x 10^{-5}</td><td>2.10 x 10^{-4}</td><td>2.10 x 10^{-4}</td><td colspan="3" rowspan="3">ND*</td><td colspan="3" rowspan="3">ND</td></tr>
<tr><td>8.78 x 10^{-5}</td><td>1.26 x 10^{-4}</td><td>1.30 x 10^{-4}</td></tr>
<tr><td>2.78 x 10^{-5}</td><td>5.20 x 10^{-5}</td><td>6.00 x 10^{-5}</td></tr>
</table>

Table S4: *Release testing criteria for the drug substance*

<table>
<tr><th colspan="2">Release Testing</th></tr>
<tr><th>Test</th><th>Method</th></tr>
<tr><td>Appearance</td><td>Visual inspection</td></tr>
<tr><td>Physical properties</td><td>pH
USP <791> / Ph. Eur. <2.2.3></td></tr>
<tr><td>Potency
Strength 1 (1E+07 PFU/mL)</td><td>Titration by Plaque forming unit counts (PFU) plus qPCR</td></tr>
<tr><td>Strength 2 (1E+09 PFU/mL)</td><td>Titration by Plaque forming unit counts (PFU) plus qPCR</td></tr>
<tr><td>Strength 3 (1E+11 PFU/mL)</td><td>Titration by Plaque forming unit counts (PFU) plus qPCR</td></tr>
<tr><td rowspan="2">Identification</td><td>PCR</td></tr>
<tr><td>PCR using Cas specific primers</td></tr>
<tr><td>Endotoxin</td><td>Chromogenic kinetic method (LAL); (USP<85>; Ph. Eur. 2.6.14)</td></tr>
<tr><td rowspan="5">Bioburden</td><td>TAMC (USP <61> / Ph. Eur. <2.6.12>)</td></tr>
<tr><td>TYMC (USP <61> / Ph. Eur. <2.6.12>)</td></tr>
<tr><td>Absence of E. coli in 1 mL (USP <62> / Ph. Eur. <2.6.13>)</td></tr>
<tr><td>Absence of bile-tolerant Gram-negative bacteria in 1 mL (USP <62> / Ph. Eur. <2.6.13>)</td></tr>
<tr><td>Absence of Staphylococcus aureus (S. aureus) in 1 mL (USP <62> / Ph. Eur. <2.6.13>)</td></tr>
</table>

	Absence of *Salmonella* in 1 mL (USP <62> / Ph. Eur. <2.6.13>)
	Absence of *Clostridia* in 1 mL (USP <62> / Ph. Eur. <2.6.13>)
	Absence of *Pseudomonas aeruginosa* in 1 mL (USP <62> / Ph. Eur. <2.6.13>)
	Absence of *Candida albicans* in 1 mL (USP <62> / Ph. Eur. <2.6.13>)

[1]Other non-target phages from SNIPR001
[2]*E. coli* HCP ELISA kit, Cygnus Technologies
[3]Benzonase ELISA kit II, Merck
[4]Total aerobic microbial count (TAMC)
[5]Total yeast and mold count (TYMC)

Table S5*: List of clinical parameters that were measured during the dosing of all four phages (α15.2, α20.4, α48.4 and α51.5) in Gottingen minipigs.*

**Measurement type**	**Measurement**
Hematology	Red blood cell count
	Hematocrit
	Hemoglobin
	Mean cell volume
	Mean corpuscular hemoglobin
	Mean corpuscular hemoglobin concentration
	Mean corpuscular volume
	Reticulocyte count
	Leukocyte count
	Neutrophil count
	Lymphocyte count
	Monocyte count
	Eosinophil count
	Platelets
Renal parameters	Albumin
	Total protein
	Albumin/Globulin (A/G) ratio
	Urea
	Creatine
	Creatine kinase (CK)
Hepatic parameters	Alanine aminotransferase (ALAT)
	Aspartate aminotransferase (AST)
	Alkaline phosphatase (ALP/BASP)
	Bilirubin
	Gamma-GT (GGT)
	Cholesterol
Electrolytes	Calcium
	Magnesium
	Phosphate
	Iron
	Sodium
	Potassium
Other parameters	C-Reactive Protein (CRP)

Table S6: Primers and gBlocks

**Name**	**Sequence (5´to 3´)**	**Comment**
**FO298**	GCCGGTGCGGTACTGGCGCTCTCTTCGTCTTTTACTGTCAACGCA GCTGAAGCGACCGAGTGAGCTAGCT	Δ*tsx*::*aadA*
**FO299**	TAACCTACTACCAGGTAACCACCCCAGCCGGTAGAGCGAACGTT	Δ*tsx*::*aadA*

	GAAGTTGCTAGCCGTCAAGCTAGCAG	
**FO302**	GGGCATTGTGGCATCTGCATCCGTACAGGCCGCAGAAATATATA ACAAAGAGCGACCGAGTGAGCTAGCT	Δ*phoE*::*aadA*
**FO303**	ATTATTAATATTCAATTTGTTATCGCTATCCAGTTGGTTGATTTTA TAATGCTAGCCGTCAAGCTAGCAG	Δ*phoE*::*aadA*
**FO304**	CGTGTTCCGTCACGGCATTTTCCGCTTGGGCACAGGATACCAGCC CGGATAGCGACCGAGTGAGCTAGCT	Δ*btuB*::*aadA*
**FO305**	GTCCTGCAGTTTGGTAGCCATAGACTGTCTCATAATCTTTGTCGA ACAGGGCTAGCCGTCAAGCTAGCAG	Δ*btuB*::*aadA*
**FO306**	GCGTAAAATCGCAGTTGTAGTAGCCACAGCGGTTAGCGGCATGT CTGTTTAGCGACCGAGTGAGCTAGCT	Δ*fhuA*::*aadA*
**FO307**	AAGCAGCCATAAGTGTTAAAGCAGCTGGCGACGTATTCACGATC GAACAGGCTAGCCGTCAAGCTAGCAG	Δ*fhuA*::*aadA*
**FO308**	AACTTCCTCTGGCGGTTGCCGTCGCAGCGGGCGTAATGTCTGCTC AGGCAAGCGACCGAGTGAGCTAGCT	Δ*lamB*::*aadA*
**FO309**	AGGTCCACTCGTCGCTGTCGCCACGACCGAAGCTGCCGCCGTTGA AATCAGCTAGCCGTCAAGCTAGCAG	Δ*lamB*::*aadA*
**FO310**	TTCTTATCGGCCTGAGCCTTTCTGGGTTCAGTTCGTTGAGCCAGGC CGAGAAAGCGACCGAGTGAGCTAGCT	Δ*tolC*::*aadA*
**FO311**	TATGACCGTTACTGGTGGTAGTGCGTGCGGATGTTTGCTGAACGA CTGGTGCTAGCCGTCAAGCTAGCAG	Δ*tolC*::*aadA*
**FO312**	GAAGCGCAATATTCTGGCAGTGATCGTCCCTGCTCTGTTAGTAGC AGGTACTGCAAAGCGACCGAGTGAGCTAGCT	Δ*ompF*::*aadA*
**FO313**	ACCCACAGCAACGGTGTCGTCTGAACCTACGCCCAGTTTGTTGTC AGAATGCTAGCCGTCAAGCTAGCAG	Δ*ompF*::*aadA*
**FO314**	TTATCTTATTGCCGCCGTAGTGAGTGGCGCTCTGGCGGTATCTGG CTGCAAGCGACCGAGTGAGCTAGCT	Δ*ompA*::*aadA*
**FO315**	AGGTAATTTCTACACGGCGGTTTTGCGCCTTACCTTCTGCGGTGCT GTTGGCTAGCCGTCAAGCTAGCAG	Δ*ompA*::*aadA*
**FO316**	GTCCCTCCTGGTCCCAGCTCTGCTGGTAGCAGGCGCAGCAAACGC TGCTGAGCGACCGAGTGAGCTAGCT	Δ*ompC*::*aadA*
**FO317**	CAGAGCTACGATGTTATCAGTGTTGATGCCAGCGTCACGAGTGAA CTGGTGCTAGCCGTCAAGCTAGCAG	Δ*ompC*::*aadA*
**FO318**	TGGCAGCCGGTGCGGT	Sanger for *tsx* deletion
**FO319**	AGCCGGTAGAGCGAACGTTG	Sanger for *tsx* deletion
**FO320**	CAGCTCTGCTGGTAGCAGG	Sanger for *ompC* deletion
**FO321**	CAGACCCAGAGCTACGATG	Sanger for *ompC* deletion
**FO322**	CGTAGTGAGTGGCGCTCTG	Sanger for *ompA* deletion
**FO323**	TTAAGGTAATTTCTACACGGCGGT	Sanger for *ompA* deletion
**FO324**	GAAGCGCAATATTCTGGCAGTG	Sanger for *ompF* deletion
**FO325**	CTACGCCCAGTTTGTTGTCAGA	Sanger for *ompF* deletion
**FO326**	CATTCTTATCGGCCTGAGCCT	Sanger for *tolC* deletion
**FO327**	AGGGTTATGACCGTTACTGGTG	Sanger for *tolC* deletion
**FO328**	TACTCTGCGCAAACTTCCTCTG	Sanger for *lamB* deletion
**FO329**	GAAGGTCCACTCGTCGCTG	Sanger for *lamB* deletion
**FO330**	CTCAGCCAAAACACTCACTGC	Sanger for *fhuA* deletion
**FO331**	GAAGGTTGCGGTTGCAACGA	Sanger for *fhuA* deletion
**FO332**	CGTGTTCCGTCACGGCATT	Sanger for *btuB* deletion
**FO333**	AGGTGTAGCTGCCAGACAAG	Sanger for *btuB* deletion
**FO334**	CACTCTGGCATTAGTGGTGATG	Sanger for *phoE* deletion
**FO335**	AATATTCAATTTGTTATCGCTATCCAGTTG	Sanger for *phoE* deletion
**FO360**	TCTCGCAGTCGCAGTGGCACTTATCTCCACCCAGGCCTGGTCGGC AGGCTGCGACCGAGTGAGCTAGCT	Δ*fadL*::*aadA*
**FO361**	TCAGAACGCGTAGTTAAAGTTAGTACCGAACAGCCAGGCTTTACC TTCAGGCTAGCCGTCAAGCTAGCAG	Δ*fadL*::*aadA*
**FO362**	TCTGCTCTCGCAGTCGCAG	Sanger for *fadL* deletion
**FO363**	GTTAGTACCGAACAGCCAGG	Sanger for *fadL* deletion
**rfaDupR**	ATTCGTGTCTGAGATTGTCTCTGACTCCATAATTCGAAGGTTACA GTTATGATCATCGTTGATATCGCTAGCTCGAGCACGTGTTGAC	Δ*rfaD*::*Sh ble*
**rfaDupR**	CCAAGACGGGCCGATCACCAGTATTTTCATGCAGAGCTCTTATGC GTCGCGATTCAGCCACGTTGTAAAACGACGGCCAGTGCCAAGC	Δ*rfaD*::*Sh ble*
**rfaFrev**	GACCGCGCCTACGTCTTACCCAAC	Sanger for *rfaD* deletion
**gB149**	tCACCTGCatatctctAGCCGAGTTCCCCGCGCCAGCGGGATAAACCG	CRISPR array cloning

	TGATTGACGGCTACGGTattaGCAGGTGcctgctccgtggtgcgggtactgtgtacaat tgactcgtgaacaggagtcgctagtcgtgcactaggcgcggaacctgcactctcaaat	
**gB150**	ctgttatcctctgtaatcgggatgcctgattcaagaccctgggtaccctCACCTGCcgatCGGTAA ACCGGCAACGTTCGAGTTCCCCGCGCCAGCGGGGATAAACCGGC TGTTAACGTACGTACcagtGCAGGTGaggctatag	CRISPR array cloning
**gB152**	ctgttatcctctgtaatcgggatgcctgattcaagaccctgggtaccctCACCTGCcgatGTACCG CGCCGCATCCGGCGAGTTCCCCGCGCCAGCGGGGATAAACCGCG GACTTAGTGCCAAAACATcagtGCAGGTGaggctatag	CRISPR array cloning
**gB153**	ctgttatcctctgtaatcgggatgcctgattcaagaccctgggtaccctCACCTGCcgatACATGG CATCGAAATTGAGTTCCCCGCGCCAGCGGGGATAAACCGcgccaaaa accccgcttcggcggggtttttccgcTTCGtgagatatGCAGGTGt	CRISPR array cloning
**gB-d2**	TCACCTGCATATCTCTGATGttgacggctagctcagtcctaggtacagtgctagcAGCC TGAGATATGCAGGTGT	J23100 cloning
**TH402**	ccttcaagtattgaatTGACtgagaccctgcagtccgAAGGGCGAATTC	pM0 cloning
**TH403**	tcagcttatattgttaatccCTCCagagaccgaattccagTTAAGGGCG	pM0 cloning
**TH556**	TCACCTGCATATCTCTTGACAAACAGGGAGGCTATTAatgGAACCT TTTAAATATATATG	*cas3* and cascade cloning
**TH558**	ACACCTGCATATCTCAATGGtggactcacaaagaaaaaacgcccggtgtgcaagaccga gcgttctgaacaaTCACAGTGGAGCCAAAGA	*cas3* and cascade cloning

REFERENCES

1. Vieira, H. L. A., Freire, P. & Arraiano, C. M. Effect of Escherichia coli Morphogene bolA on Biofilms. *Appl Environ Microb* 70, 5682–5684 (2004).

2. Datsenko, K. A. & Wanner, B. L. One-step inactivation of chromosomal genes in Escherichia coli K-12 using PCR products. *Proc National Acad Sci* 97, 6640–6645 (2000).

3. Hantke, K. Compilation of Escherichia coli K-12 outer membrane phage receptors - their function and some historical remarks. *Fems Microbiol Lett* 367, (2020).
