# Supplementary material for: Engineered phage with antibacterial CRISPR–Cas selectively reduce E. coli burden in mice
Source: Nat Biotechnol. 2023 May 4;42(2):265–74. doi: 10.1038/s41587-023-01759-y (PMC10869271; doi:10.1038/s41587-023-01759-y)
Supplement: Supplementary file 2 — Reporting Summary [file 41587_2023_1759_MOESM2_ESM.pdf]

nature research

Corresponding author(s): Morten Sommer

Last updated by author(s): Mar 14, 2023

Reporting Summary

Nature Research wishes to improve the reproducibility of the work that we publish. This form provides structure for consistency and transparency in reporting. For further information on Nature Research policies, see our Editorial Policies and the Editorial Policy Checklist.

Statistics

For all statistical analyses, confirm that the following items are present in the figure legend, table legend, main text, or Methods section.

n/a	Confirmed	
☐	☒	The exact sample size (*n*) for each experimental group/condition, given as a discrete number and unit of measurement
☐	☒	A statement on whether measurements were taken from distinct samples or whether the same sample was measured repeatedly
☐	☒	The statistical test(s) used AND whether they are one- or two-sided<br>*Only common tests should be described solely by name; describe more complex techniques in the Methods section.*
☒	☐	A description of all covariates tested
☐	☒	A description of any assumptions or corrections, such as tests of normality and adjustment for multiple comparisons
☐	☒	A full description of the statistical parameters including central tendency (e.g. means) or other basic estimates (e.g. regression coefficient) AND variation (e.g. standard deviation) or associated estimates of uncertainty (e.g. confidence intervals)
☐	☒	For null hypothesis testing, the test statistic (e.g. *F*, *t*, *r*) with confidence intervals, effect sizes, degrees of freedom and *P* value noted<br>*Give P values as exact values whenever suitable.*
☒	☐	For Bayesian analysis, information on the choice of priors and Markov chain Monte Carlo settings
☒	☐	For hierarchical and complex designs, identification of the appropriate level for tests and full reporting of outcomes
☐	☒	Estimates of effect sizes (e.g. Cohen's *d*, Pearson's *r*), indicating how they were calculated

Our web collection on statistics for biologists contains articles on many of the points above.

Software and code

Policy information about availability of computer code

Data collection	Figures and key statistics were generated using R version 4.1.0. For figure generation the following packages were used: RcolorBrewer v. 1.1-2, ape v. 5.5, ggsignif v. 0.6.2, ggpubr v. 0.4.0, matrixStats 0.59, reshape2 v. 1.4.4, ggimage v. 0.3.0, here v. 1.0.1, purr v. 0.3.4, ggtree63 v. 3.0.2, systemfonts v. 1.0.2, Cairo v. 1.5-12.2, cowplot v. 1.1.1, reaxxl v. 1.3.1, and ggplot2 v.3.3.3, openxlsx, v. 4.2.3, patchwork v. 1.1.1, dplyr v. 1.0.7, and ggh4x v. 0.2.3. Averages and standard deviations are calculated after transforming the values to the scale shown on a given figure, e.g. when a log10 scale is used, the averages and standard deviations are calculated after log10 transformation. The synteny plot was then generated using a custom Python (v 3.7.10) script, using the drawSvg library (v 1.9.0).
Data analysis	All code needed to produce this study is available at https://github.com/sniprbiome/SNIPR001_paper.

For manuscripts utilizing custom algorithms or software that are central to the research but not yet described in published literature, software must be made available to editors and reviewers. We strongly encourage code deposition in a community repository (e.g. GitHub). See the Nature Research guidelines for submitting code & software for further information.

Data

Policy information about availability of data

All manuscripts must include a data availability statement. This statement should provide the following information, where applicable:

- Accession codes, unique identifiers, or web links for publicly available datasets
- A list of figures that have associated raw data
- A description of any restrictions on data availability

All data and results that were generated during this study is deposited at https://github.com/sniprbiome/SNIPR001_paper. Additional data are available in the Article, Online methods and Supplementary tables. The MLST database was downloaded on July 1, 2021, from the MLST2 repository (https://bitbucket.org/genomicepidemiology/mlst_db/src/master/). For annotation of the CAP sequences the following tools and datasets were used ResFinder 4.1 (https://

cge.cbs.dtu.dk/services/ResFinder), VirulenceFinder-2.0 (https://cge.cbs.dtu.dk/services/VirulenceFinder/), PHASTER Prophage/Virus DB (https://phaster.ca/). To reproduce the results, no further data is needed.
Phage genome sequences are deposited at Genbank under access numbers OQ067373 - 76

Field-specific reporting

Please select the one below that is the best fit for your research. If you are not sure, read the appropriate sections before making your selection.

☒ Life sciences ☐ Behavioural & social sciences ☐ Ecological, evolutionary & environmental sciences

For a reference copy of the document with all sections, see nature.com/documents/nr-reporting-summary-flat.pdf

Life sciences study design

All studies must disclose on these points even when the disclosure is negative.

Sample size	No explicit sample size calculations were performed since the strains assayed where part of the SENTRY surveillance program. The JMI panel comprises of 382 strain E. coli clinical collection obtained from JMI Laboratories (North Liberty, IA, USA). These strains were isolated from patients with bloodstream infections hospitalized in hematology and oncology units across four different regions (Asia-Pacific 54 isolates, Europe 161 isolates, Latin America 26 isolates, and North America 141 isolates), sourced through the SENTRY Antimicrobial Surveillance Program (2018–2020), which is composed of a network of more than 150 medical centers in more than 28 countries worldwide (https://www.jmilabs.com/sentry-surveillance-program).
Data exclusions	no exclusions were performed
Replication	all experiments contain at least two biological replicates and for each method the number of technical replicates are stated. The SNIPR001 assay against the 382 strains was performed in duplicate and the duplicate results are explicitly shown in Figure 5B by the two dots.
Randomization	not relevant for this work as all 382 E. coli isolates were exposed to SNIPR001 and received the same treatment.
Blinding	not relevant for this work as the 382 E. coli isolates were exposed to SNIPR001. More specifically for counting of phage titers, phage lysates or the equal volume mix of SNIPR001 CAPs were serially diluted 10-fold in SM buffer or PBS, respectively. Bacterial lawns were prepared by adding 100 or 300 µL of bacterial overnight culture to 3 or 10 mL of 0.5% top agar (containing Ca2+ and Mg2+), which was vortexed briefly and poured onto a round or square LB plate. Five µl of the dilution series of test phages were then spotted onto lawns and left to dry at room temperature with an open lid prior to incubation at 37ºC overnight. The strains b52, b2479 and b17 were used as controls of the assay and included in each round of assays.<br>The next day, results were assessed (Extended Data Table 4). In this assay, a susceptible strain is defined as one producing plaques that are countable in PFU/mL as well as one without visible plaques but demonstrating impairment of bacterial growth (i.e., lysis zones). Coverage defines the percentage of the total number of susceptible strains. Images of all plates were recorded. Figures illustrating efficiency of plating results first had titers log10 transformed and then standard deviances and averages were calculated subsequently. The clinical panels and control strains were tested in two independent experiments.

Reporting for specific materials, systems and methods

We require information from authors about some types of materials, experimental systems and methods used in many studies. Here, indicate whether each material, system or method listed is relevant to your study. If you are not sure if a list item applies to your research, read the appropriate section before selecting a response.

Materials & experimental systems

n/a	Involved in the study	
☒	☐	Antibodies
☒	☐	Eukaryotic cell lines
☒	☐	Palaeontology and archaeology
☐	☒	Animals and other organisms
☒	☐	Human research participants
☒	☐	Clinical data
☒	☐	Dual use research of concern

Methods

n/a	Involved in the study	
☒	☐	ChIP-seq
☒	☐	Flow cytometry
☒	☐	MRI-based neuroimaging

Animals and other organisms

Policy information about studies involving animals; ARRIVE guidelines recommended for reporting animal research

Laboratory animals	female CD-1® IGS mice (approximately 6–7 weeks of age upon arrival) from Charles River (Freiburg, Germany). And female Göttingen

Laboratory animals	minipigs (approximately 4-7 months of age upon arrival) from Ellegaard Göttingen minipigs A/S, Denmark was used for tolerability and kinetic studies.
Wild animals	did not involve wild animals
Field-collected samples	no field samples were collected
Ethics oversight	All procedures were conducted in accordance with guidelines from the Danish Animal Experiments Inspectorate, Ministry of Environment and Food of Denmark and in accordance with the institutional license (BioAdvice, animal license no. 2015-15-0201-00540).

Note that full information on the approval of the study protocol must also be provided in the manuscript.
